# TRPC6 Attenuates Cortical Astrocytic Apoptosis and Inflammation in Cerebral Ischemic/Reperfusion Injury

**DOI:** 10.3389/fcell.2020.594283

**Published:** 2021-02-02

**Authors:** Lu Liu, Manli Chen, Kun Lin, Xuwu Xiang, Jing Yang, Yueying Zheng, Xiaoxing Xiong, Shengmei Zhu

**Affiliations:** ^1^Department of Anesthesiology, The First Affiliated Hospital, Zhejiang University School of Medicine, Hangzhou, China; ^2^Department of Neurosurgery, Renmin Hospital of Wuhan University, Wuhan, China

**Keywords:** apoptosis, astrocytes, Ca^2+^, inflammation, ischemic stroke, NF-κB, TRPC6

## Abstract

Transient receptor potential canonical 6 (TRPC6) channel is an important non-selective cation channel with a variety of physiological roles in the central nervous system. Evidence has shown that TRPC6 is involved in the process of experimental stroke; however, the underlying mechanisms remain unclear. In the present study, the role of astrocytic TRPC6 was investigated in an oxygen–glucose deprivation cell model and middle cerebral artery occlusion (MCAO) mouse model of stroke. HYP9 (a selective TRPC6 agonist) and SKF96365 (SKF; a TRPC antagonist) were used to clarify the exact functions of TRPC6 in astrocytes after ischemic stroke. TRPC6 was significantly downregulated during ischemia/reperfusion (IR) injury in cultured astrocytes and in cortices of MCAO mice. Application of HYP9 *in vivo* alleviated the brain infarct lesion, astrocytes population, apoptosis, and interleukin-6 (IL-6) and IL-1β release in mouse cortices after ischemia. HYP9 dose-dependently inhibited the downregulation of TRPC6 and reduced astrocytic apoptosis, cytotoxicity and inflammatory responses in IR insult, whereas SKF aggravated the damage *in vitro*. In addition, modulation of TRPC6 channel diminished IR-induced Ca^2+^ entry in astrocytes. Furthermore, decreased Ca^2+^ entry due to TRPC6 contributed to reducing nuclear factor kappa light chain enhancer of activated B cells (NF-κB) nuclear translocation and phosphorylation. Overexpression of astrocytic TRPC6 also attenuated apoptosis, cytotoxicity, inflammatory responses, and NF-κB phosphorylation in modeled ischemia in astrocytes. The results of the present study indicate that the TRPC6 channel can act as a potential target to reduce both inflammatory responses and apoptosis in astrocytes during IR injury, subsequently attenuating ischemic brain damage. In addition, we provide a novel view of stroke therapy by targeting the astrocytic TRPC6 channel.

## Introduction

The transient receptor potential (TRP) channels are cation-permeable membrane proteins with common structural features of six transmembrane segments. Based on the homology of amino acid sequences, the TRP superfamily can be subdivided into seven subfamilies: TRPC (canonical), TRPV (vanilloid), TRPM (melastatin), TRPA (ankyrin), TRPN (*Drosophila* NOMPC), TRPP (polycystin), and TRPML (mucolipin) (Venkatachalam and Montell, 2007). Among the channels, the TRPC channel, which is most closely related to *Drosophila* TRPs, is widely distributed in different tissues and governs the fate and functions of various cell types (Curcic et al., 2019). TRPCs are broadly expressed in brain, lung, heart, kidney, liver, spleen, and other organs in mammalian animals like human, mouse, rat, and rabbit (Montell, 2001; Venkatachalam and Montell, 2007). In terms of similarity to amino acid sequences and function, TRPC members can be further classified into four subgroups: TRPC1, TRPC2, TRPC4/5, and TRPC3/6/7 (Wang et al., 2020a).

The TRPC6 channel is emerging as an important target for the control of Ca^2+^ currents in a wide range of disorders, including immune-mediated diseases, pulmonary arterial hypertension, atherosclerosis, and central nervous system (CNS)-related diseases, such as autism spectrum disorders, glioma, depression, traumatic brain injuries, seizure, Alzheimer's disease, and ischemic stroke (Hamid and Newman, 2009; Ding et al., 2010; Du et al., 2010; Griesi-Oliveira et al., 2015; Kim and Kang, 2015; Zhang et al., 2015, 2016; Pochwat et al., 2018; Ramirez et al., 2018; Chen et al., 2019). TRPC6 is abundantly expressed in various anatomical regions of the CNS, such as the cerebellum, hippocampus, middle frontal gyrus, and cortex (Riccio et al., 2002; Du et al., 2010). As a regulator of Ca^2+^ influx, TRPC6 is involved in neuronal survival, synapse formation, neuronal nerve-growth-cone guidance, and sensory transduction (Li et al., 2005; Jia et al., 2007; Zhou et al., 2008; Quick et al., 2012). Dysfunction of the TRPC6 channel may trigger a series of downstream events and neurobiological disorders.

Ischemic stroke is a life-threatening condition caused by a vascular embolism due to cardiac events, artery-to-artery embolism, or *in-situ* small artery disease (Hankey, 2017). Several underlying mechanisms, including excitotoxicity, ionic imbalance, oxidative and nitrative stress, inflammation, and apoptosis, are involved in the pathophysiological process of cerebral ischemia (Khoshnam et al., 2017). Ca^2+^ overload has a critical role and initiates the ischemic cascade during brain ischemia/reperfusion (IR) injury. Increasing evidence indicates an important role of TRPC6 in cerebral IR injury (Liu et al., 2020). TRPC6 is identified on cortical neurons and astrocytes, and is downregulated in neurons after brain ischemic injury (Du et al., 2010; Guo et al., 2017; Qu et al., 2017; Shirakawa et al., 2017). Notably, maintaining the TRPC6 protein level in neurons improves neuronal survival and behavioral performance, thus alleviating ischemic brain damage (Du et al., 2010; Guo et al., 2017). However, the roles of the TRPC6 channel in mouse cortical astrocytes following IR injury have not been evaluated.

In the present study, the specific effects of astrocytic TRPC6 on ischemic stroke were investigated. TRPC6 protein expression in primary mouse astrocytes was downregulated following IR injury. Inhibition of TRPC6 downregulation via HYP9 or TRPC6 overexpression protected astrocytes and the brain against IR insults. These results indicate that the TRPC6 channel contributes to neuroprotection in cerebral ischemia by promoting astrocyte survival. Furthermore, this study provides therapeutic evidence for the treatment of ischemic stroke by targeting the TRPC6 channel.

## Materials and Methods

### Animals

C57BL/6J male mice 8–10 weeks of age were purchased from Shanghai SLAC Laboratory Animal Co., Ltd. (Shanghai, China). All animal experiments were approved by the Animal Care Committee of the First Affiliated Hospital at Zhejiang University. Mice were housed in polypropylene cages and maintained at 25 ± 1°C under 12 h light/12 h dark cycles with free access to rodent chow and water. Mice were randomly allocated to each group before any treatment. Proper anesthetic procedures were used to ensure that the mice did not suffer unnecessarily during or after the experimental procedure. A total of 82 mice were used in this study (including 10 mice that died).

### *In vivo* Model of Focal Cerebral Ischemia

Focal cerebral ischemia was induced by middle cerebral artery occlusion (MCAO). Animals were anesthetized with 1% sodium pentobarbital (75–100 mg/kg, intraperitoneal injection); body temperature was maintained at 37 ± 0.5°C during the operation using a heating pad. Transient MCAO was generally performed as previously reported (Lin et al., 2013a). Briefly, the left common carotid artery was exposed to separate the internal carotid artery (ICA) and the external carotid artery (ECA). Next, a 6-0 monofilament nylon suture (RWD Life Science Co., LTD, Shenzhen, China) with a rounded tip was inserted through the exposed left ICA and advanced into the middle cerebral artery. After 1 h of occlusion, the filament was gently withdrawn to allow reperfusion. At 24 h after reperfusion, animals were sacrificed and the brain tissues were obtained for future assays.

### Intracerebroventricular Injection

Mouse intracerebroventricular injection was performed using a stereotaxic instrument (RWD Life Science Co., Ltd., Shenzhen, China) with a micro-syringe pump under anesthesia. Drugs or vehicle (5 μL in total) were slowly injected (1 μL/min) into the left ventricle at a depth of 2.5 mm below the brain surface, 1.0 mm lateral and 0.5 mm posterior to the bregma. After injection, animals were allowed to recover from anesthesia under a heating pad.

### Cell Culture

As previously reported(Shen et al., 2016), primary astrocytes from cerebral cortices of 0–1-day-old post-natal C57BL/6 mice were isolated under sterile conditions. Astrocytes were grown in culture medium [Dulbecco's modified Eagle's medium (DMEM) containing 10% fetal bovine serum and 1% penicillin/streptomycin]. Dissociated cortical cells were seeded onto poly-D-lysine (PDL, 10 μg/mL; # P7405; Sigma-Aldrich St. Louis, MO, USA)-coated T-75 flasks (Costar; Corning Inc., Corning, NY, USA) at a density of three cortices per flask and incubated at 37°C with 5% CO_2_ in a humidified incubator. The medium was changed every other day. After 8–10 days, confluent cultures were shaken (250 rpm at 37°C) for 12 h to reduce microglial contamination. Then, purified astrocytes were cultured in medium supplemented with 20 μM cytosine-1-β-D-arabinofuranosid (Sigma-Aldrich) for the next 2–3 days. The remaining attached cells were digested with 0.25% trypsin (#25200056; Gibco, Grand Island, NY, USA) and then reseeded at a density of 0.03–0.05 × 10^6^/cm^2^ in PDL-coated six-well plates at 37°C in an incubator with 5% CO_2_. After 5–7 days when the astrocytes reached 90–95% confluence, cells were prepared for subsequent experiments. More than 95% of cells in culture were astrocytes (results not shown).

### Oxygen-Glucose Deprivation and Re-oxygenation (OGD/R)

OGD/R experiments were established to mimic the *in vitro* condition of IR injury. The cultures were incubated for 1–4 h in DMEM medium without glucose in a humidified incubator chamber at 37°C with 95% N_2_ and 5% CO_2_. Subsequently, the astrocytes were returned to the original culture conditions and then incubated under normoxic conditions for the next 24 h.

### Drugs and Experimental Groups

The TRPC6-specific agonist HYP9 (Leuner et al., 2010) (#H9791; Sigma-Aldrich), dissolved in dimethyl sulfoxide (DMSO; the final maximum DMSO concentration was <0.05%), was used to determine the role of TRCP6 in astrocytes following IR injury. To verify the optimum concentration, 0, 1, 5, 10, 15, 20, or 30 μM HYP9 was preincubated *in vitro*. SKF96365 (SKF; #ab120280; Abcam, Cambridge, MA, USA) was originally recognized as a major inhibitor of TRPC channels (Singh et al., 2010). In the present study, SKF (dissolved in deionized water) at 0, 0.05, 0.5, 5, 10, 20, 30, or 40 μM concentration was used to treat primary mouse astrocytes. The *in vitro* groups consisted of eight subgroups: (1) control group (CON + Naive); (2) control combined with vehicle (DMSO) group (CON + Vehicle); (3) control combined with HYP9 (15 μM) group (CON + HYP9); (4) control combined with SKF (30 μM) group (CON + SKF); (5) OGD/R group (OGD + Naive); (6) OGD/R combined with vehicle (DMSO) group (OGD + Vehicle); (7) OGD/R combined with HYP9 (15 μM) group (OGD + HYP9); (8) OGD/R combined with SKF (30 μM) group (OGD + SKF).

**Figure 1:**
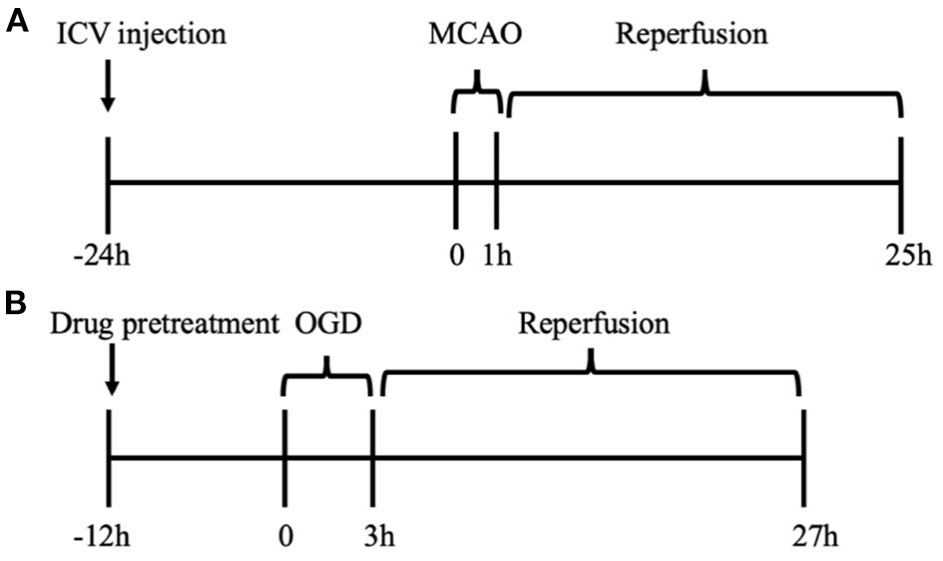
Experiment paradigm. **(A)** *In vivo* cohort for MCAO model of ischemic stroke in mice. The drugs were administrated into left ventricles 24 h before MCAO. **(B)** *In vitro* cohort for OGD/R experiment in primary mouse astrocytes. After 12 h pre-incubation of drugs, OGD/R was developed in cultured astrocytes.

Animals were randomly assigned into eight groups before any procedure as follows: (1) sham operation group (sham + Naive) (*n* = 9); (2) sham operation combined with vehicle (DMSO) group (sham + Vehicle) (*n* = 3); (3) sham operation combined with HYP9 (5 μg) group (sham + HYP9) (*n* = 3); (4) sham operation combined with SKF (20 μg) group (sham + SKF) (*n* = 3); (5) MCAO operation group (MCAO + Naive) (*n* = 18); (6) MCAO operation combined with DMSO group (MCAO + Vehicle) (*n* = 12); (7) MCAO operation combined with HYP9 (5 μg) group (MCAO + HYP9) (*n* = 12); (8) MCAO operation combined with SKF (20 μg) group (MCAO + SKF) (*n* = 12). Figure 1 shows the experiment paradigm.

### Lentivirus Infection

Lentivirus vectors were chemically synthesized by Obio Technology (Shanghai) Corp., Ltd. To overexpress astrocytic TRPC6, we infected primary astrocytes with lentiviruses carrying FLAG-tagged full-length mCherry-WT-TRPC6 (WT-TRPC6) or mCherry-WT-TRPC6-null (Vehicle1). Besides, to knock down TRPC6 in primary astrocytes, lentiviruses carrying mCherry-shRNA-TRPC6 (sh-TRPC6) or mCherry-shRNA-TRPC6-null (Vehicle2) were used to infect primary cortical astrocytes. Targeted sequences for TRPC6 and Vehicle2 siRNAs are: GCTTGCCAACATTGAGAAA and TTCTCCGAACGTGTCACGT, respectively. Astrocytes at 70% confluence were infected with lentiviruses according to the vendor's protocol. After 72–96 h lentiviral infection, astrocytes were used for subsequent experiments. The efficiency of infections was detected by western bolts.

### TTC Staining

Animals were sacrificed at 24 h after reperfusion. The infarct volume was tested based on 2,3,5-triphenyltetrazolium chloride (TTC) staining. The brains were sectioned into 2-mm coronal slices using a brain matrix. The brain slices were incubated in 2% TTC solution staining for 20 min at 37°C and then fixed in 4% paraformaldehyde (PFA). The ischemic volume was quantified using ImageJ software (version 2.0). The infarct percentage was shown as the ratio of the ischemic area to the entire slice area.

### Analysis of Cell Viability and Lactate Dehydrogenase (LDH) Release

An LDH release quantification kit (#11644793001; Sigma-Aldrich) was used to evaluate cell viability. The cell-free supernatant was obtained by centrifugation at ~250 × g for 10 min. Subsequently, the supernatant was incubated in working solutions in the dark for 1 h at room temperature (~22°C). The absorbance of the samples was measured at 490 nm using a microplate reader (SpectraMax i3x; Molecular Devices, Sunnyvale, CA, USA). To determine the cytotoxicity percentage, all groups were compared with the control groups.

### Flow Cytometry

The flow cytometry measurement of astrocyte apoptosis and Ca^2+^ concentration was performed using a BD flow cytometer (BD Biosciences, San Jose, CA, USA). Astrocytes were digested with trypsin. The harvested cells were washed with phosphate-buffered saline (PBS) twice before incubating in buffer containing 5 μL of fluorescein isothiocyanate-annexin V and 5 μL of propidium iodide or 0.5 μg/ml DAPI for 15 min at room temperature in the dark. Subsequently, the cells were detected and analyzed.

The Ca^2+^ concentration was also measured using the BD flow cytometer. Cortical astrocytes were loaded with 2 μM Fluo-4 AM (#F14217; Invitrogen, Carlsbad, CA, USA) in culture medium for 1 h at 37°C in an incubator with 5% CO_2_. Next, the medium was changed to Hank's balanced salt solution (#14025092; Gibco) and the astrocytes were incubated for another 30 min at 37°C. Then, the astrocytes were collected and analyzed using flow cytometry.

### Western Blot

Western blot analysis was performed as previously described with slight modifications (Li et al., 2020). Samples (20–40 μg) were separated on 12% gels and transferred onto 0.45-μm polyvinylidene difluoride membranes (#IPVH00010; Millipore, Billerica, MA, USA). Membranes were blocked with 5% skim milk for 1 h and then incubated with the following primary antibodies at 4°C overnight: TRPC6 (1;1,000; #21403; SAB Biotherapeutics, Sioux Falls, SD, USA), caspase-3 (1:1,000; #ab214430; Abcam, Cambridge, MA, USA), NF-κB (1:1,000; #6956S; Cell Signaling Technology), phospho-NF-κB (1:1,000; #3033S, Cell Signaling Technology), IL-1β (1:2,000; #ab9722; Abcam), Flag (1:1000; # F1804, Sigma-Aldrich), β-actin (1:2,000; #60008-1-Ig; Proteintech, Rosemont, IL, USA). Then, the membranes were incubated for 1 h with secondary antibodies (1:8,000; #SA00001-1 and #SA00001-2; Proteintech). Protein-specific signals were visualized using the Bio-Rad ChemiDoc™ MP imaging system (Hercules, CA, USA).

### Immunofluorescence

Brain tissues were cut into 20 μm slices using a cryostat (#CM1950; Leica, Wetzlar, Germany). Tissues were blocked in 5% goat serum containing 0.3% Triton X-100 for 1 h at 37°C and then incubated with the following primary antibodies overnight at 4°C: GFAP (1:400; #80788S and #3670S; Cell Signaling Technology), TRPC6 (1:100; #ab62461; Abcam). Next, the slices were washed with PBS and incubated with secondary fluorescent antibodies (1:100; #SA00013-1, SA00013-2, SA00013-3, or SA00013-4; Proteintech) for 1 h at room temperature. In addition, 4′,6-iamidino-2-phenylindole, dihydrochloride (DAPI; 1 μg/mL; #4083; Cell Signaling Technology) staining was used to visualize the nuclei. Images were acquired using a confocal laser-scanning microscope (Nikon A1 Ti, 600× magnification; Leica TCS SP8, 400× magnification).

Astrocytes plated on glass coverslips 12 mm in diameter were collected and fixed in 4% PFA for 15 min at room temperature. Subsequently, the fixed cells were permeabilized with 0.1% Triton X-100 for 15 min at room temperature followed by blocking in 10% goat serum at 37°C for 30 min. Before incubation with secondary fluorescent antibodies, the astrocytes were labeled with the following primary antibodies: GFAP (1:400; #80788S and #3670S; Cell Signaling Technology), NF-κB (1:100; #6956S; Cell Signaling Technology). Next, DAPI (1 μg/mL) was used to stain nuclei. Specific fluorescent signals were tested using a confocal microscope (Leica TCS SP8, 630× or 1,890× magnification).

### Enzyme-Linked Immunosorbent Assay

The release of interleukin-6 (IL-6) and IL-1β from cortices and cultured astrocytes was quantified using enzyme-linked immunosorbent assay (IL-6, #70-EK206HS; IL-1β, #70-EK201BHS-96; MULTI SCIENCES, Hangzhou, China). The experiments were performed according to the manufacturer's instructions. The final absorbance was detected at 450 nm and the background correction was set at 570 nm on a microplate reader (SpectraMax i3x; Molecular Devices).

### Statistical Analyses

Data are presented as the means ± standard error of the mean (SEM) of at least three independent analyses. SPSS ver. 25.0 software (IBM Corp., Armonk, NY, USA) and GraphPad Prism 8.0 software (GraphPad Software, San Diego, CA, USA) were used for statistical analyses. One-way analysis of variance with Tukey's test for *post-hoc* analysis were performed. *P*-value < 0.05 was considered statistically significant.

## Results

### TRPC6 in Mouse Cortical Astrocytes Is Downregulated After Ischemia

In several studies, TRPC6 in neurons reportedly declines after cerebral ischemia, and maintaining the expression of neuronal TRPC6 protein contributes to neuronal survival and reduced cerebral ischemic insult (Du et al., 2010; Lin et al., 2013a; Guo et al., 2017). However, the role of the astrocytic TRPC6 channel after stroke remains unclear.

**Figure 2:**
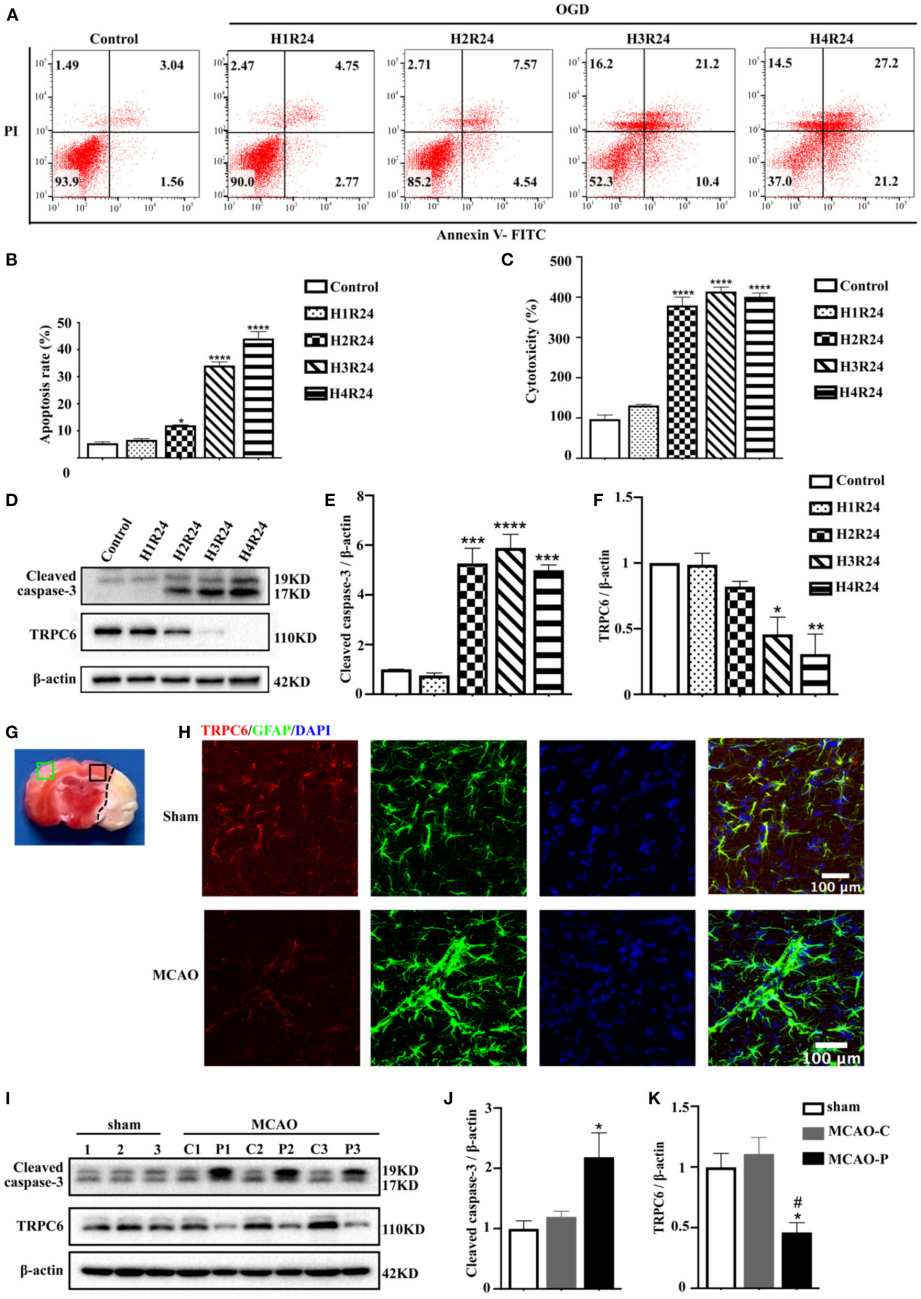
TRPC6 in the cortical astrocytes is downregulated in ischemia. **(A)** Cultured astrocytes were exposed or not to OGD/R, and their apoptosis and death was detected by FITC/PI flow cytometry analysis. The percentage of apoptosis in scatter plots was presented as the proportion of apoptotic cells vs. total cell population (H1R24: 1 h OGD followed by 24 h reperfusion; H2R24: 2 h OGD followed by 24 h reperfusion; H3R24: 3 h OGD followed by 24 h reperfusion; H4R24: 4 h OGD followed by 24 h reperfusion). **(B)** Bar chart showed the statistical results of apoptosis rate measured by flow cytometry in each group in three independent experiments. Data were shown as mean ± SEM (*n* = 3). **(C)** LDH assay was used to measure the cytotoxicity of astrocytes in control and OGD groups. Data were shown as mean ± SEM (*n* = 4). **(D)** The protein level of TRPC6 and cleaved caspase-3 in astrocytes were tested by western blot analysis. β-actin was used as a loading control. **(E,F)** Quantifications of cleaved caspase-3 and TRPC6 protein levels shown in **(D)**. Data were shown as mean ± SEM (*n* = 3). **(G)** Brain slices with TTC staining. Green rectangle: contralateral cortex; Black rectangle: peri-infarct cortex. **(H)** Representative images of the cortex after sham or MCAO operation (peri-infarct cortex) double-stained with the TRPC6 (Red) and GFAP (Green) antibodies (Scar bar: 100 μm; 600× magnification). **(I)** Mice were exposed to sham operation or MCAO procedure (*n* = 3). The expressions of TRPC6 and cleaved caspase-3 in sham group (left hemisphere) and in contralateral (C) or peri-infarct (P) cortex were detected by western blotting. β-actin was used as a loading control. The number “1,” “2,” “3” represents cortices in each group were extracted form three different mice. **(J,K)** Quantifications of cleaved caspase-3 and TRPC6 protein levels shown in **(I)**. Data were shown as mean ± SEM (*n* = 3). MCAO-C, contralateral cortices in MCAO mice; MCAO-P, peri-infarct cortices in MCAO mice. *p < 0.05, **p < 0.01, ***p < 0.001, ****p < 0.0001 (all comparisons to control group or sham group). ^#^*p* < 0.05 (comparisons to MCAO-C group).

**Figure 3:**
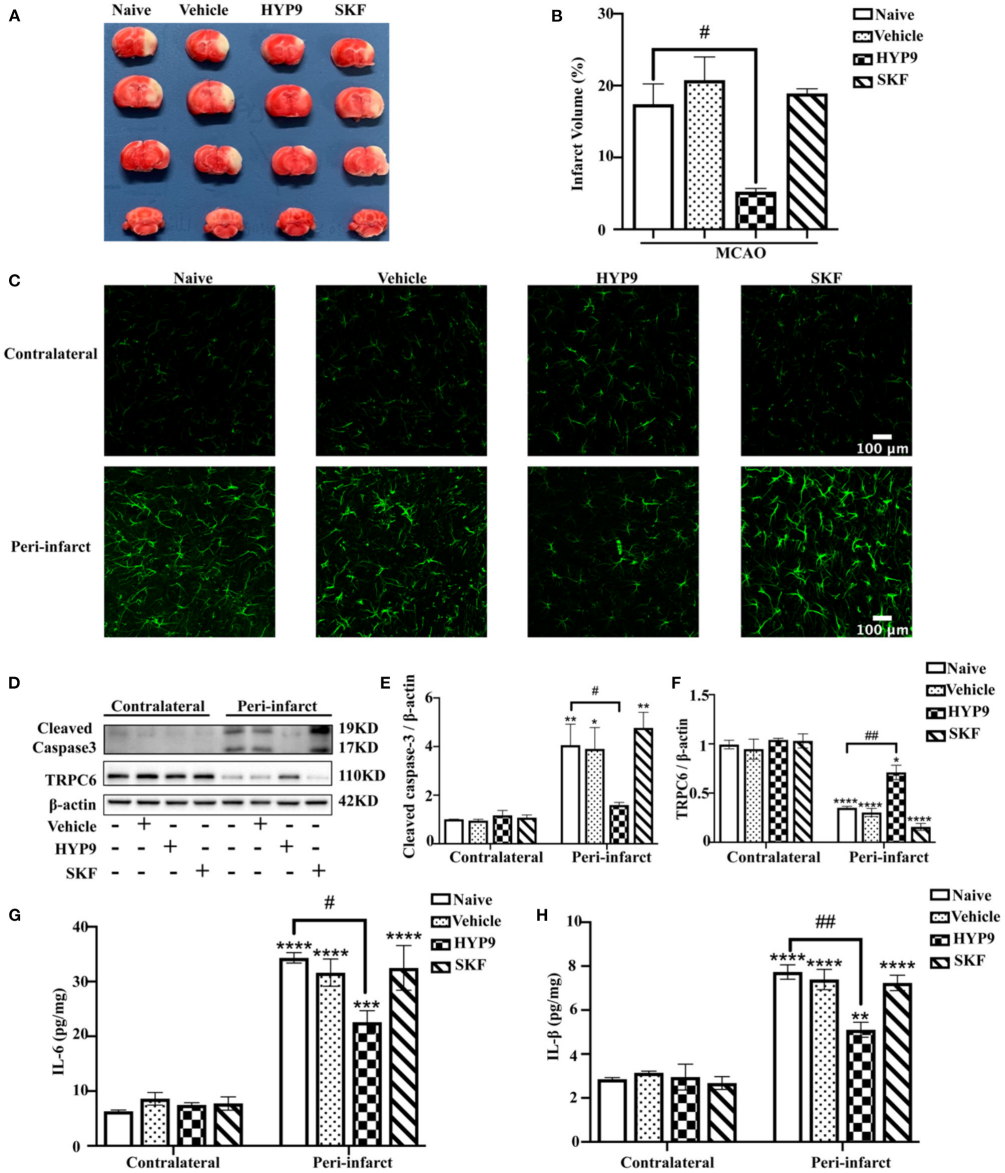
Reducing IR-mediated TRPC6 downregulation with HYP9 alleviates ischemic damage in mice. Vehicle (DMSO), 5 μg HYP9, or 20 μg SKF were injected into left ventricles of mice 24 h before sham or MCAO operation. **(A)** TTC-stained cerebral slices indicated the infarct size in mice. **(B)** Quantification of infarct volumes after ischemia. Data were shown as mean ± SEM (*n* = 3). ^#^*p* < 0.05 vs. MCAO + Naive group. **(C)** Representative images of astrocytes population with GFAP (green) staining in contralateral or peri-infarct cortices in MCAO mice (Scar bar: 100 μm; 400× magnification). **(D)** Western blot detecting TRPC6 and cleaved caspase-3 in mouse cortices. β-actin was used as a loading control. **(E,F)** Quantifications of cleaved caspase-3 and TRPC6 protein levels shown in **(D)**. Data were shown as mean ± SEM (*n* = 3). **(G,H)** The release of IL-6 and IL-1β was determined by ELISA assay. Data were shown as mean ± SEM (*n* = 3). *p < 0.05, **p < 0.01, ***p < 0.001, ****p < 0.0001 vs. MCAO + Naïve group in contralateral cortices; ^#^*p* < 0.05, ^##^*p* < 0.01 vs. MCAO + Naive group in peri-infarct area.

Astrocytes have provided important insights into ischemic stroke (Liu and Chopp, 2016). To confirm the vital functions of the astrocytic TRPC6 channel in cerebral ischemia, we established an *in vitro* model of stroke in primary mouse astrocytes using an OGD/R experiment. Astrocytes were exposed to 1–4 h of OGD followed by 24 h reperfusion. The apoptosis rate was elevated and cell viability was decreased in astrocytes following hypoxia injury (Figures 2A–C). The TRPC6 protein levels in astrocytes after OGD/R were significantly downregulated (Figures 2D,F). Conversely, the expression of the apoptosis-specific biomarker, cleaved caspase-3, was gradually increased after stroke compared with the control groups (Figures 2D,E). As shown in Figures 2A–D, 3 h OGD and 24 h reperfusion triggered appropriate hypoxic damage in astrocytes; thus, the subsequent *in vitro* OGD/R experiments were performed under this condition.

The *in vivo* stroke model is mimicked by MCAO in mice. Immunofluorescence signals showed that astrocytic TRPC6 was downregulated in the ischemic penumbra (Figures 2G,H). In parallel with the *in vitro* results, the TRPC6 protein level in peri-infarct areas was decreased compared with that in contralateral cortices (Figures 2I,K). In addition, after ischemic stroke, the cleaved caspase-3 expression in the ipsilateral hemisphere was upregulated compared with the contralateral hemisphere (Figures 2I,J). Furthermore, immunofluorescence signals showed that astrocytic TRPC6 was downregulated in the ischemic penumbra (Figure 2H).

These results indicate that IR injury-induced changes in TRPC6 channel protein levels may have important functions in brain ischemia.

### Reducing IR-Mediated TRPC6 Downregulation With HYP9 Alleviates Ischemic Insults in the Mouse Cortex

Consequently, to determine the role of TRPC6 in an *in vivo* model of ischemic stroke, intraventricular administration of HYP9 or SKF was performed in mice. MCAO resulted in increased infarct size and pro-inflammatory cytokine release compared with the sham groups (Figure 3). Pretreatment with HYP9 alleviated cortical injury, reduced infarct volume (Figures 3A,B), astrocytic population (Figure 3C), cleaved caspase-3 expression (Figures 3D,E), and inhibited pro-inflammatory cytokine (IL-6 and IL-1β) generation after MCAO (Figures 3G,H). However, the effects of SKF on an animal model of stroke regarding infarction volume, astrocytic population, cleaved caspase-3 protein level, and cytokine generation were not significantly different. In conclusion, the results show that application of the TRPC6 agonist HYP9 can alleviate IR-induced cerebral injury in an animal model of stroke.

### Dose-Dependent HYP9 Inhibition of TRPC6 Downregulation in Astrocytes Promotes Astrocyte Survival During IR Injury

**Figure 4:**
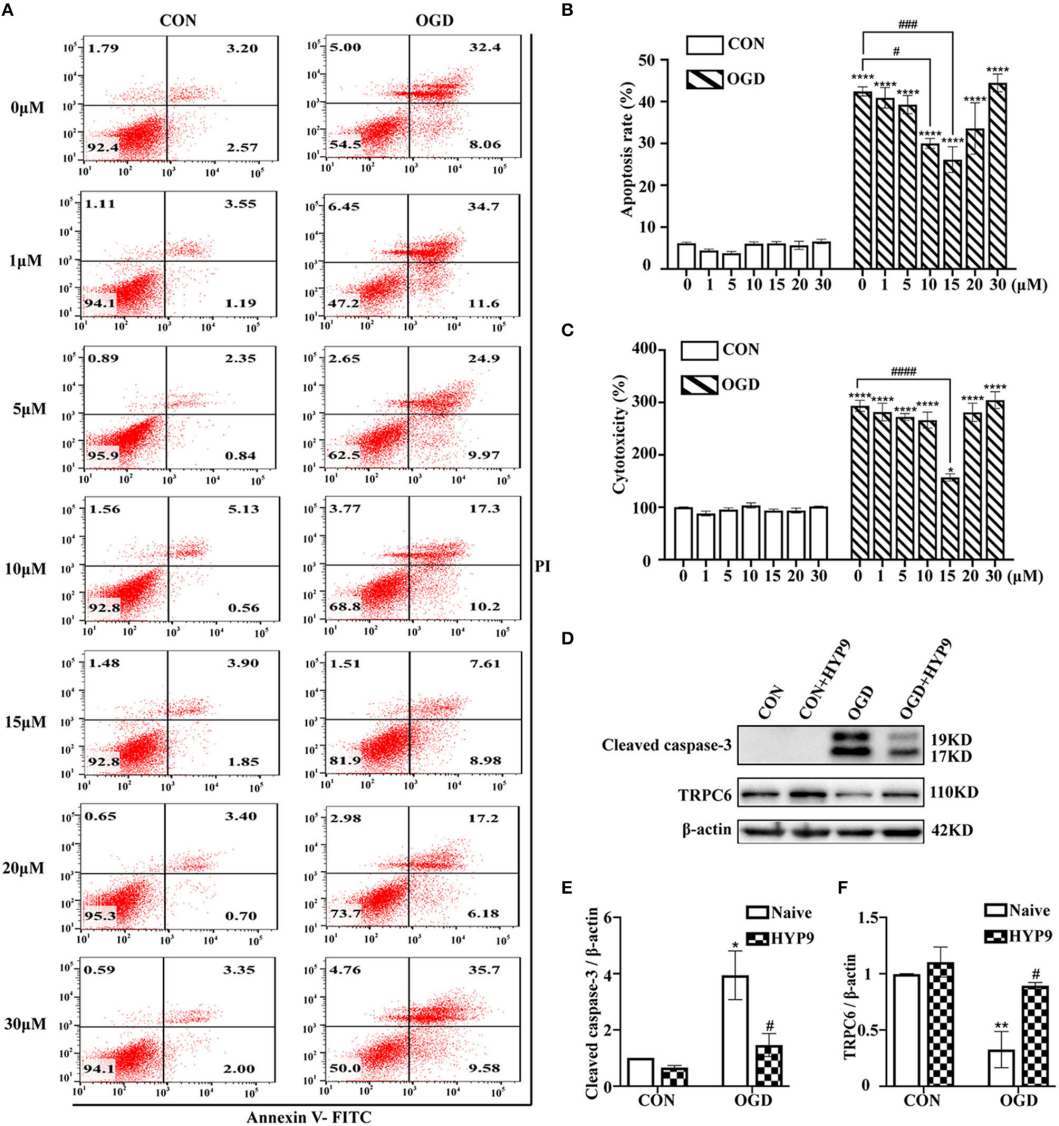
Inhibition of TRPC6 downregulation by HYP9 protects astrocytes from ischemic damage. Astrocytes were pre-incubated with 0, 1, 5, 10, 15, 20, and 30 μM HYP9 for 12 h, and then subjected to control condition (CON) or 3 h OGD and 24 h reperfusion (OGD). **(A)** Representative scatter plots of astrocytic apoptosis measured by Annexin-V FITC/PI flow cytometry in each group. **(B)** Cell apoptosis rate was analyzed by Annexin-V FITC/PI flow cytometry, and the data were shown as mean ± SEM (*n* = 4). **(C)** Quantification of OGD/R-mediated cell cytotoxicity by LDH assay. Data were shown as mean ± SEM (*n* = 4). **(D)** Immunoblots for TRPC6 and cleaved caspase-3 of the extracts from CON or OGD-treated cortical astrocytes acquiring 15 μM HYP9 or not. β-actin was used as a loading control. **(E,F)** Quantifications of cleaved caspase-3 and TRPC6 protein levels shown in **(D)**. Data were shown as mean ± SEM (*n* = 3). *p < 0.05, **p < 0.01, ****p < 0.0001 vs. CON (HYP9 = 0 μM) group; ^#^*p* < 0.05, ^###^*p* < 0.001, ^####^*p* < 0.0001 vs. OGD (HYP9 = 0 μM) group.

HYP9 is a specific TRPC6 channel activator (Leuner et al., 2010). Treatment with HYP9 (0, 1, 5, 10, 15, 20, or 30 μM) was used to verify whether TRPC6 channel degradation is responsible for IR-induced astrocyte insults. Astrocytes were pre-incubated with different concentrations of HYP9 for 12 h in a CO_2_ chamber and then exposed to normal or OGD/R conditions. Application of 15 μM HYP9 contributed to a significant decrease in OGD/R-induced astrocyte apoptosis and cytotoxicity (Figures 4A–C). Western blot results showed that HYP9 (15 μM) suppressed the downregulation of TRPC6 and decreased cleaved caspase-3 protein levels in primary mouse astrocytes in the OGD group (Figures 4D–F). These results indicate that maintaining the protein level of the TRPC6 channel in astrocytes with HYP9 diminishes OGD/R-induced astrocyte apoptosis and improves cell vitality.

### TRPC Antagonist SKF Aggravates Astrocyte Damage Dose-Dependently in the OGD/R Experiment

**Figure 5:**
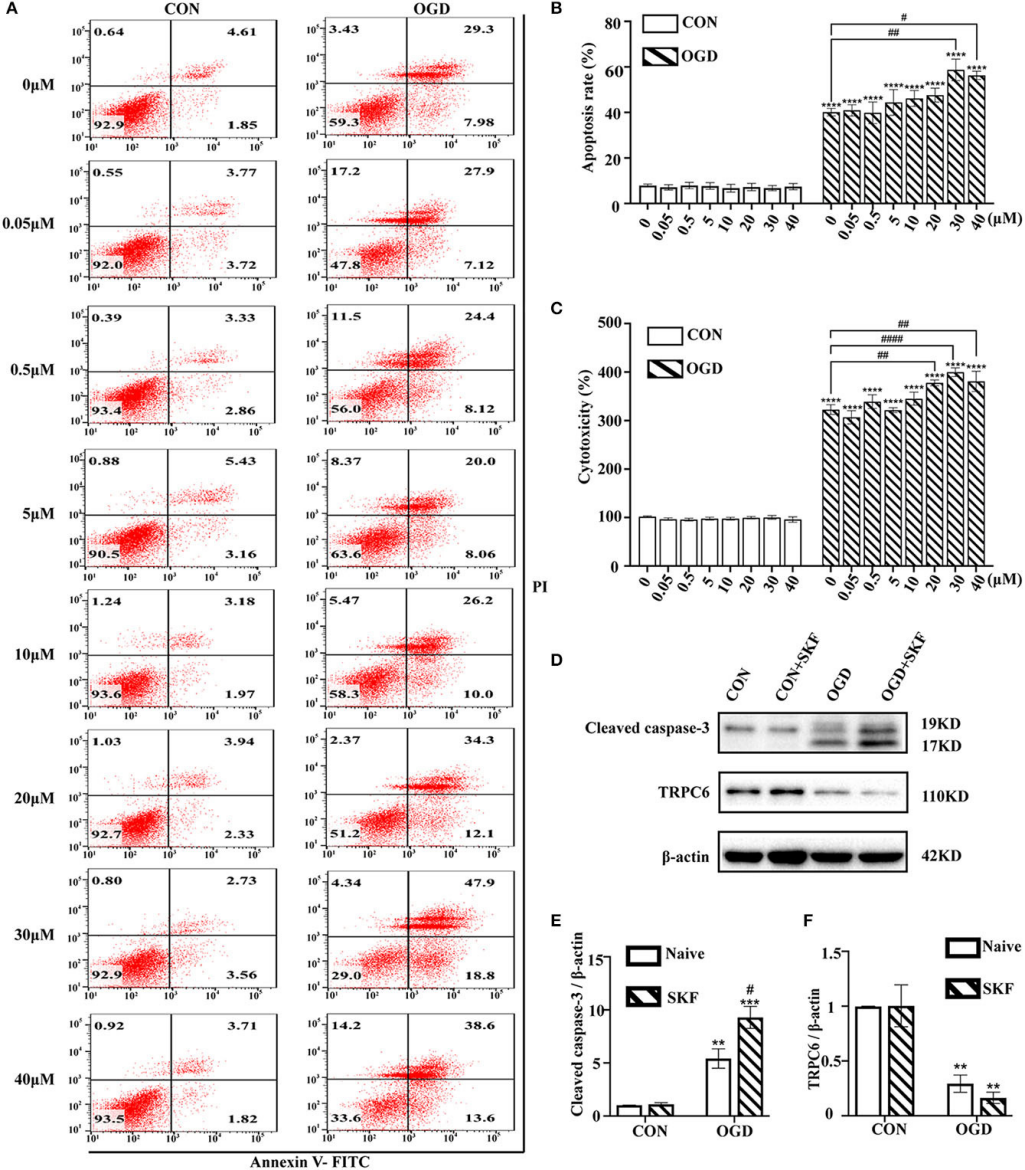
TRPC antagonist SKF aggravates OGD/R induced astrocytes insults. Astrocytes were pre-incubated with 0, 0.05, 0.5, 5, 10, 20, 30, and 40 μM SKF for 12 h, and then subjected to control condition (CON) or 3 h OGD and 24 h reperfusion (OGD). **(A)** Representative scatter plots of astrocytic apoptosis measured by Annexin-V FITC/PI flow cytometry in each group. **(B)** Cell apoptosis rate was analyzed by Annexin-V FITC/PI flow cytometry, and the data were shown as mean ± SEM (*n* = 4). **(C)** Quantification of OGD/R-mediated cell cytotoxicity by LDH assay. Data were shown as mean ± SEM (*n* = 4). **(D)** Immunoblots for TRPC6 and cleaved caspase-3 of the extracts from CON or OGD-treated cortical astrocytes acquiring 30 μM SKF or not. β-actin was used as a loading control. **(E,F)** Quantifications of cleaved caspase-3 and TRPC6 protein levels shown in **(D)**. Data were shown as mean ± SEM (*n* = 3). **p < 0.01, ***p < 0.001, ****p < 0.0001 vs. CON (SKF = 0 μM) group; ^#^*p* < 0.05, ^##^*p* < 0.01, ^####^*p* < 0.0001 vs. OGD (SKF = 0 μM) group.

Next, whether blockage of the TRPC6 channel by SKF results in increased astrocyte damage after OGD/R was investigated. Cultured astrocytes were pretreated with 0, 0.05, 0.5, 5, 10, 20, 30, or 40 μM SKF for 12 h before the OGD/R experiment. As shown in Figures 5A,B, the apoptosis of astrocytes was markedly increased in the OGD group supplemented with 30 or 40 μM SKF compared with the OGD group without SKF pretreatment. In addition, the effects of SKF on astrocyte cytotoxicity in the control and OGD groups were investigated using the LDH assay. Notably, IR injury-mediated astrocyte cytotoxicity was elevated after pre-incubation with 20, 30, or 40 μM SKF in OGD groups (Figure 5C). In addition to the increased cleaved caspase-3 expression, the OGD/R-induced degradation of TRPC6 protein was not rescued after treatment with SKF (Figures 5D–F). Taken together, pretreatment of cultured astrocytes with 30 or 40 μM SKF aggravates apoptosis and cytotoxicity in OGD groups.

### TRPC6 Protects Astrocytes Against IR Injury in Modeled Ischemia

**Figure 6:**
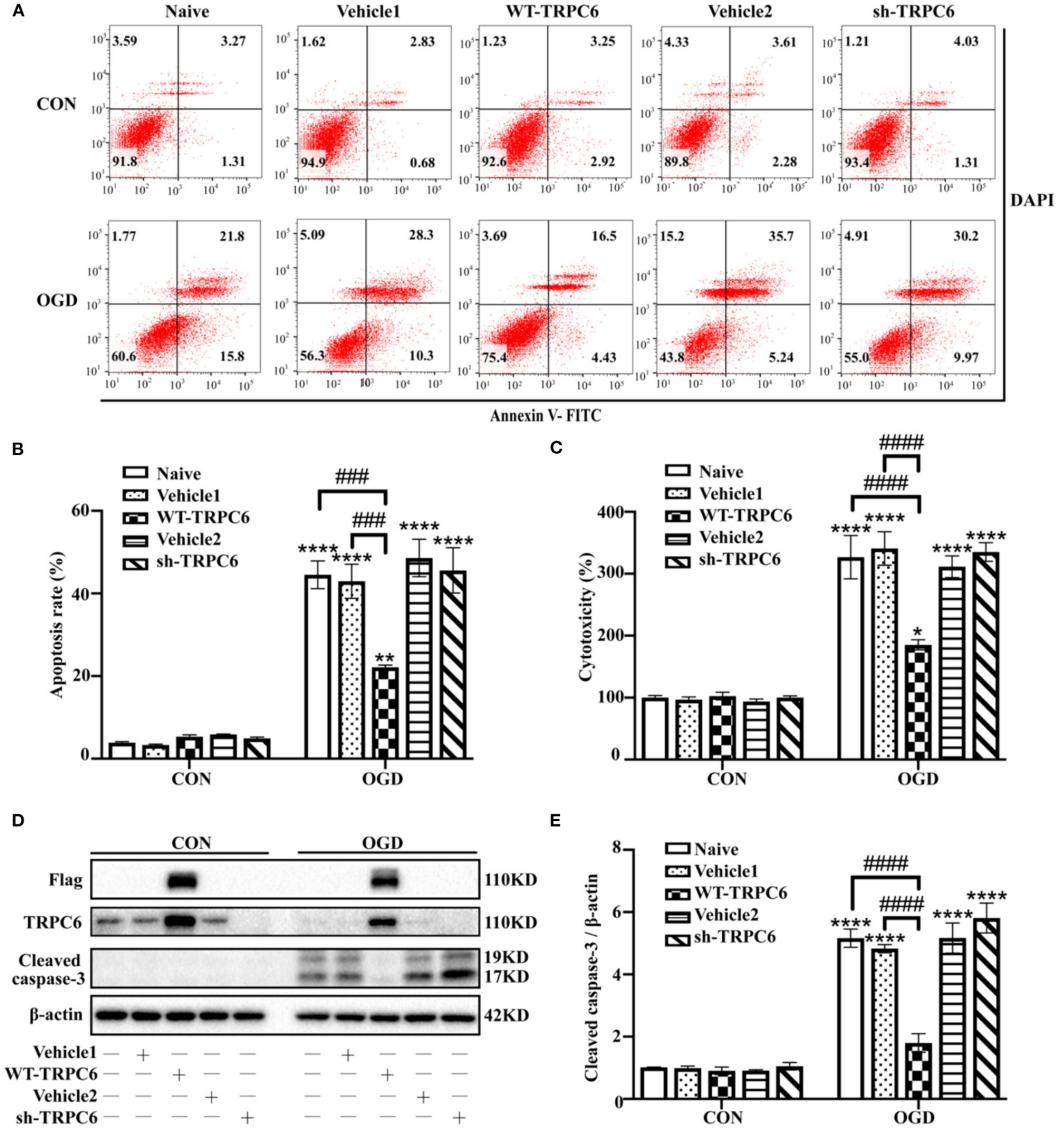
TRPC6 protects astrocytes against IR injury in modeled ischemia. Astrocytes were infected with lentiviruses carrying mCherry-WT-TRPC6-null (Vehicle1), FLAG-tagged full-length mCherry-WT-TRPC6 (WT-TRPC6), mCherry-shRNA-TRPC6-null (Vehicle2), or mCherry-shRNA-TRPC6 (sh-TRPC6), and then exposed to control condition (CON) or 3 h OGD and 24 h reperfusion (OGD). **(A)** Representative scatter plots of astrocytic apoptosis measured by Annexin-V FITC/DAPI flow cytometry in each group. **(B)** Cell apoptosis rate was analyzed by Annexin-V FITC/DAPI flow cytometry, and the data were shown as mean ± SEM (*n* = 4). **(C)** Quantification of OGD/R-mediated cell cytotoxicity by LDH assay. Data were shown as mean ± SEM (*n* = 4). **(D)** Immunoblots for Flag, TRPC6, and cleaved caspase-3 of the extracts from control or OGD-treated cortical astrocytes acquiring Vehicle1, WT-TRPC6, Vehicle2, or sh-TRPC6 vectors. β-actin was used as a loading control. **(E)** Quantification of cleaved caspase-3 protein levels shown in **(D)**. Data were shown as mean ± SEM (*n* = 3). *p < 0.05, **p < 0.01, ****p < 0.0001 vs. CON + naive group; ^###^*p* < 0.001, ^####^*p* < 0.0001 vs. OGD + naive or OGD + Vehicle1 group.

To further clarify the protective role of astrocytic TRPC6 during IR injury, we then infected primary cortical astrocytes with lentivirus vectors to overexpress (WT-TRPC6) or knock down (sh-TRPC6) TRPC6. The efficiency of lentiviral infections was shown in Figure 6D. OGD/R notably decreased the protein levels of TRPC6 compared with that in control groups in astrocytes (Figure 6D). Overexpression of TRPC6 strikingly reduced OGD/R-induced astrocyte apoptosis (Figures 6A,B), cytotoxicity (Figure 6C), and cleaved caspase-3 protein levels (Figures 6D,E). However, knocking down TRPC6 via sh-TRPC6 did not exacerbate IR injury-mediated apoptosis, cytotoxicity or cleaved caspase-3 protein levels in astrocytes (Figure 6). In general, TRPC6 attenuates astrocytes IR injury in modeled ischemia *in vitro*.

### Maintaining the Protein Level of the TRPC6 Channel in Astrocytes Alleviates Astrocytic Inflammatory Responses

**Figure 7:**
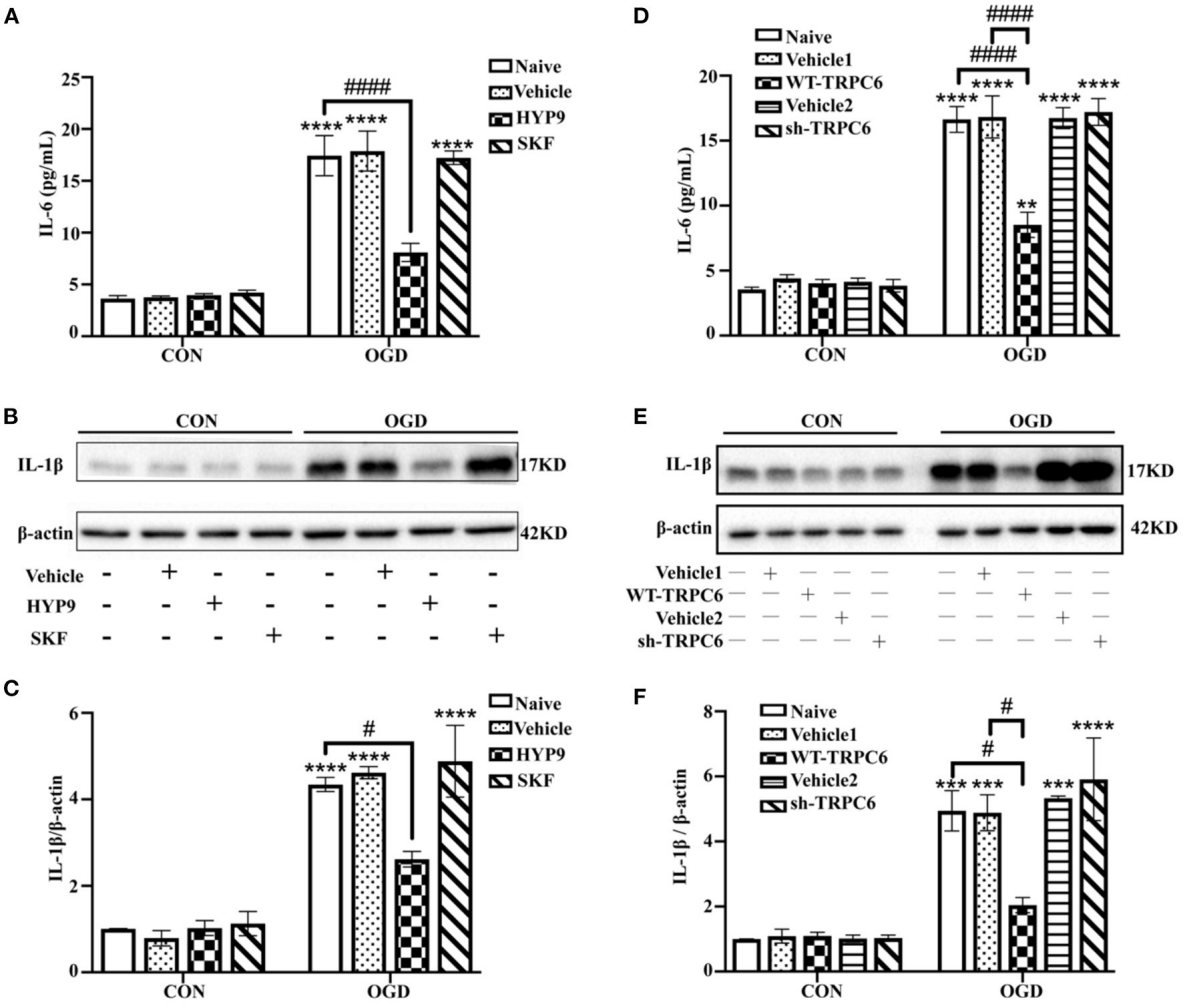
Maintaining the protein level of TRPC6 attenuates astrocytic inflammatory responses. Astrocytes were pre-incubated with DMSO (as vehicle, the final DMSO concentration was 0.025%), 15 μM HYP9 or 30 μM SKF for 12 h, and then exposed to control condition (CON) or 3 h OGD and 24 h reperfusion (OGD). **(A)** The protein level of IL-6 was determined by ELISA assay. Data were shown as mean ± SEM (*n* = 4). **(B)** The expression of pro-inflammatory cytokine IL-1β in cortical astrocytes was measured using western blotting. β-actin was used as a loading control. **(C)** Quantification of IL-1β protein levels shown in **(B)**. Data were shown as mean ± SEM (*n* = 3). Astrocytes were infected with lentiviruses carrying Vehicle1, WT-TRPC6, Vehicle2, or sh-TRPC6, and then exposed to control condition (CON) or 3 h OGD and 24 h reperfusion (OGD). **(D)** The protein level of IL-6 was determined by ELISA assay. Data were shown as mean ± SEM (*n* = 4). **(E)** The expression of pro-inflammatory cytokine IL-1β in cortical astrocytes was measured using western blotting. β-actin was used as a loading control. **(F)** Quantification of IL-1β protein levels shown in **(E)**. Data were shown as mean ± SEM (*n* = 3). **p < 0.01, ***p < 0.001, ****p < 0.0001 vs. CON + Naive group; ^#^*p* < 0.05, ^####^*p* < 0.0001 vs. OGD + Naive or OGD + Vehicle1 group.

Astrocytic inflammatory response also has a critical effect on regulating the progress and prognosis of ischemic stroke (Cekanaviciute and Buckwalter, 2016; Deng et al., 2018). In addition to cell apoptosis and vitality, the expression of the pro-inflammatory cytokines IL-6 and IL-1β were measured to confirm the effects of HYP9 and SKF on ischemic astrocytic inflammation. The IL-6 and IL-1β protein levels were significantly increased in the OGD groups compared with the control groups. Notably, HYP9 and WT-TRPC6 reduced the elevated level of IL-6 and IL-1β in astrocytes in the OGD groups (Figures 7A–F).

The data show that inhibition of TRPC6 downregulation with HYP9 or WT-TRPC6 reduces inflammatory responses in astrocytes after OGD/R.

### Inhibiting TRPC6 Downregulation Suppresses Intracellular Ca^2+^ Overload in Primary Astrocytes

**Figure 8:**
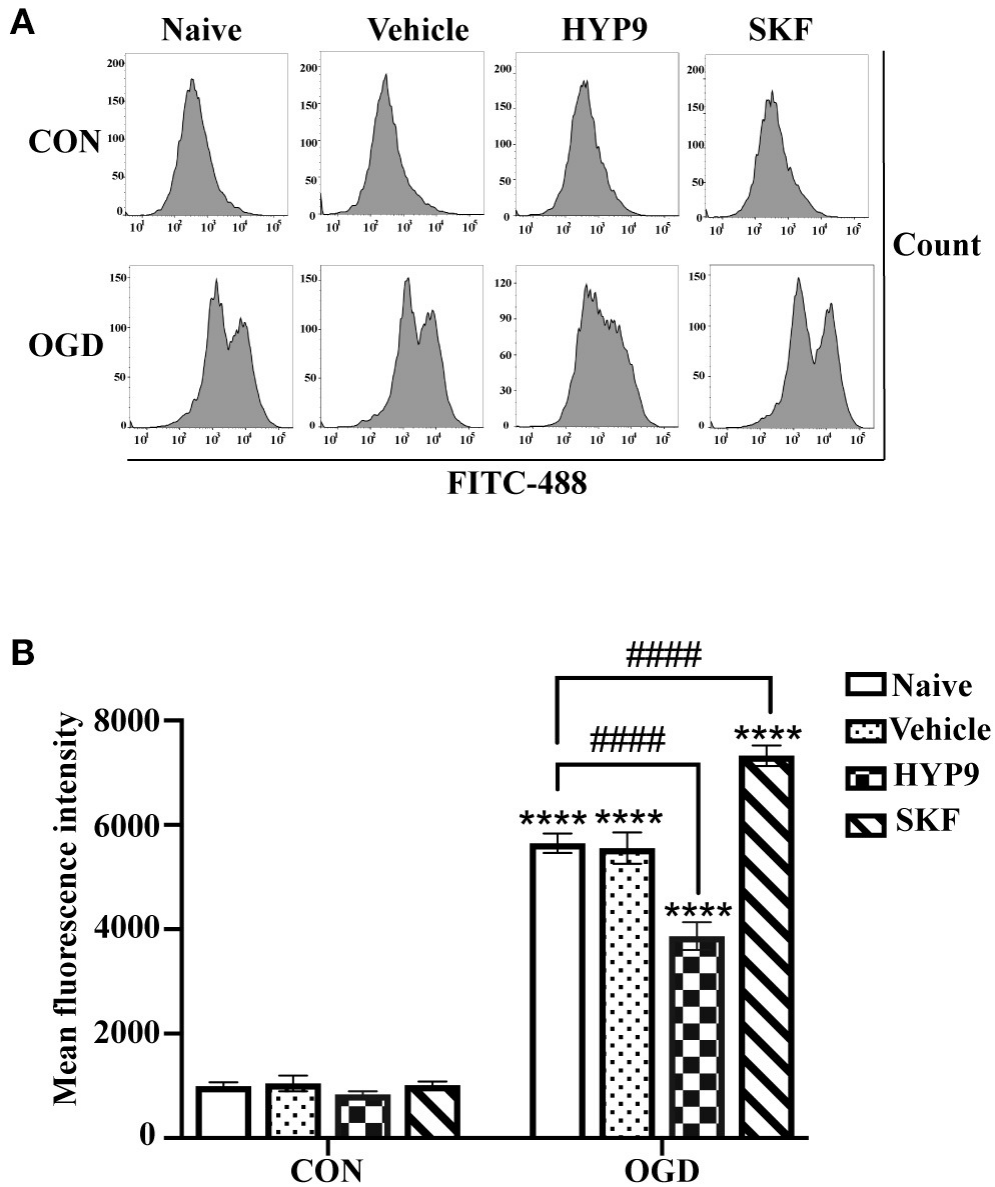
TRPC6-specific agonists HYP9 suppresses intracellular Ca^2+^ overload in primary astrocytes. Astrocytes were pre-incubated with Vehicle (DMSO, the final concentration was 0.025%), 15 μM HYP9 or 30 μM SKF for 12 h, and then exposed to control condition (CON) or 3 h OGD and 24 h reperfusion (OGD). **(A)** Representative charts of average fluorescence intensity. The level of intracellular Ca^2+^ in astrocytes was measured by Ca^2+^ fluorescence probe Fluo-4 AM through FITC-488 flow cytometry. **(B)** Quantification of Ca^2+^ concentration in astrocytes by flow cytometry. Data were shown as mean ± SEM (*n* = 4). ****p < 0.0001 vs. CON + Naive group; ^####^*p* < 0.0001 vs. OGD + Naive group.

The above reported results indicate the TRPC6 channel is closely associated with apoptosis, vitality, and inflammatory response in cultured cortical astrocytes exposed to IR damage. As a Ca^2+^ channel, TRPC6 modulates the concentration of intracellular Ca^2+^ ([Ca^2+^]_i_) (Wang et al., 2020a). To understand the potential mechanism of TRPC6 channel against cerebral ischemic insults in astrocytes, the effects of HYP9 and SKF on [Ca^2+^]_i_ in astrocytes was measured. Consistent with previous research (Li et al., 2015; Rakers and Petzold, 2017), OGD/R dramatically increased astrocytic [Ca^2+^]_i_ compared with control conditions (Figures 8A,B). Notably, application of HYP9 statistically reduced the elevated [Ca^2+^]_i_ in the OGD group (Figures 8A,B). In addition, SKF raised [Ca^2+^]_i_ in ischemic astrocytes (Figures 8A,B). These results indicate that astrocytic TRPC6 channel triggers a variety of protective cellular responses by inhibiting brain ischemia-induced Ca^2+^ overload.

### TRPC6 Diminishes NF-κB Nuclear Translocation and Phosphorylation

**Figure 9:**
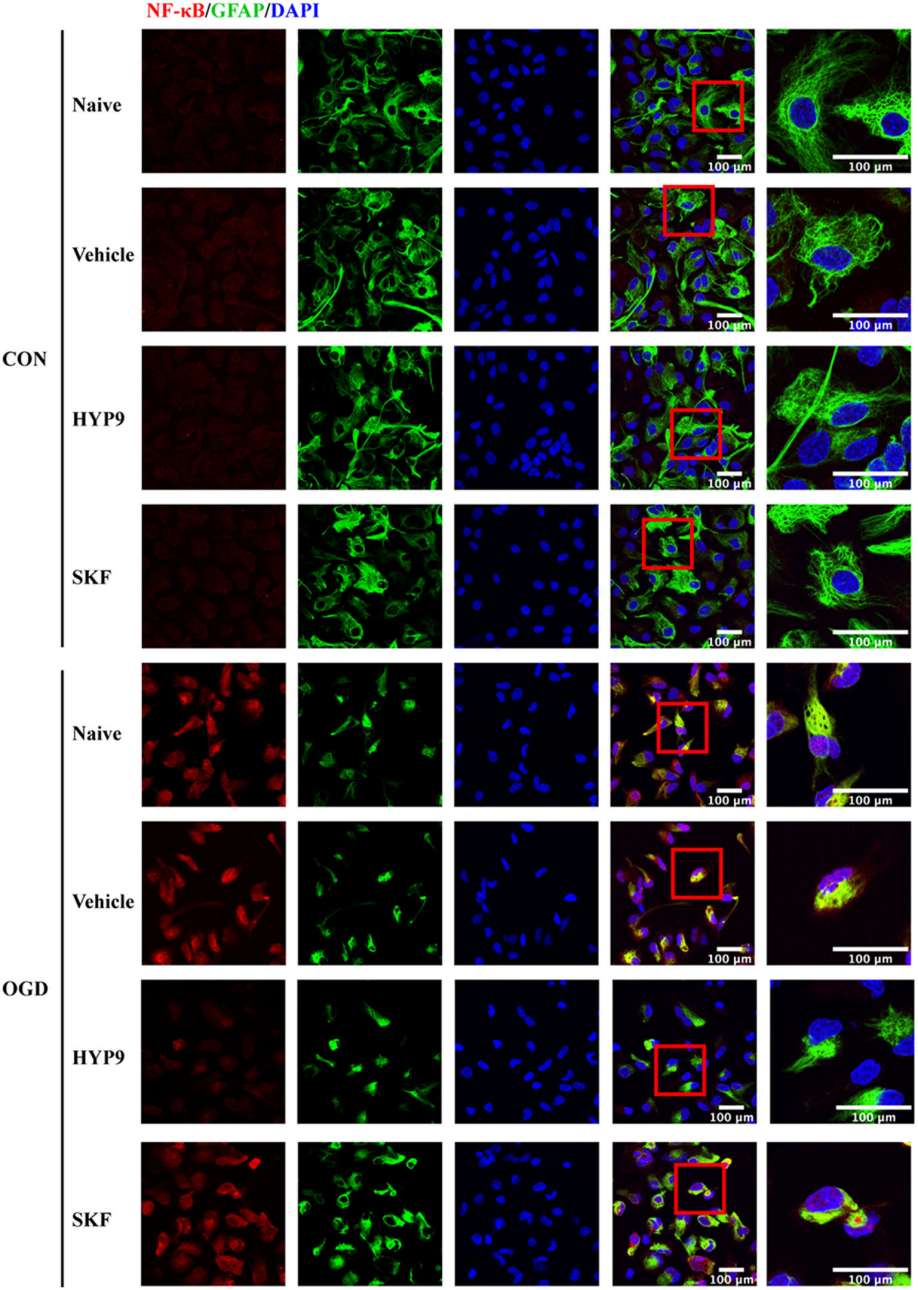
TRPC6 diminishes NF-κB nuclear translocation in astrocytes after ishcemia. Astrocytes were pre-incubated with Vehicle (DMSO, the final concentration is 0.025%), 15 μM HYP9 or 30 μM SKF for 12 h, and then expose to control condition (CON) or 3 h OGD and 24 h reperfusion (OGD). Representative images of cortical astrocytes after OGD/R double-stained with the NF-κB (red) and GFAP (green) antibodies (Scar bar: 100 μm; 630× or 1,890× magnification).

**Figure 10:**
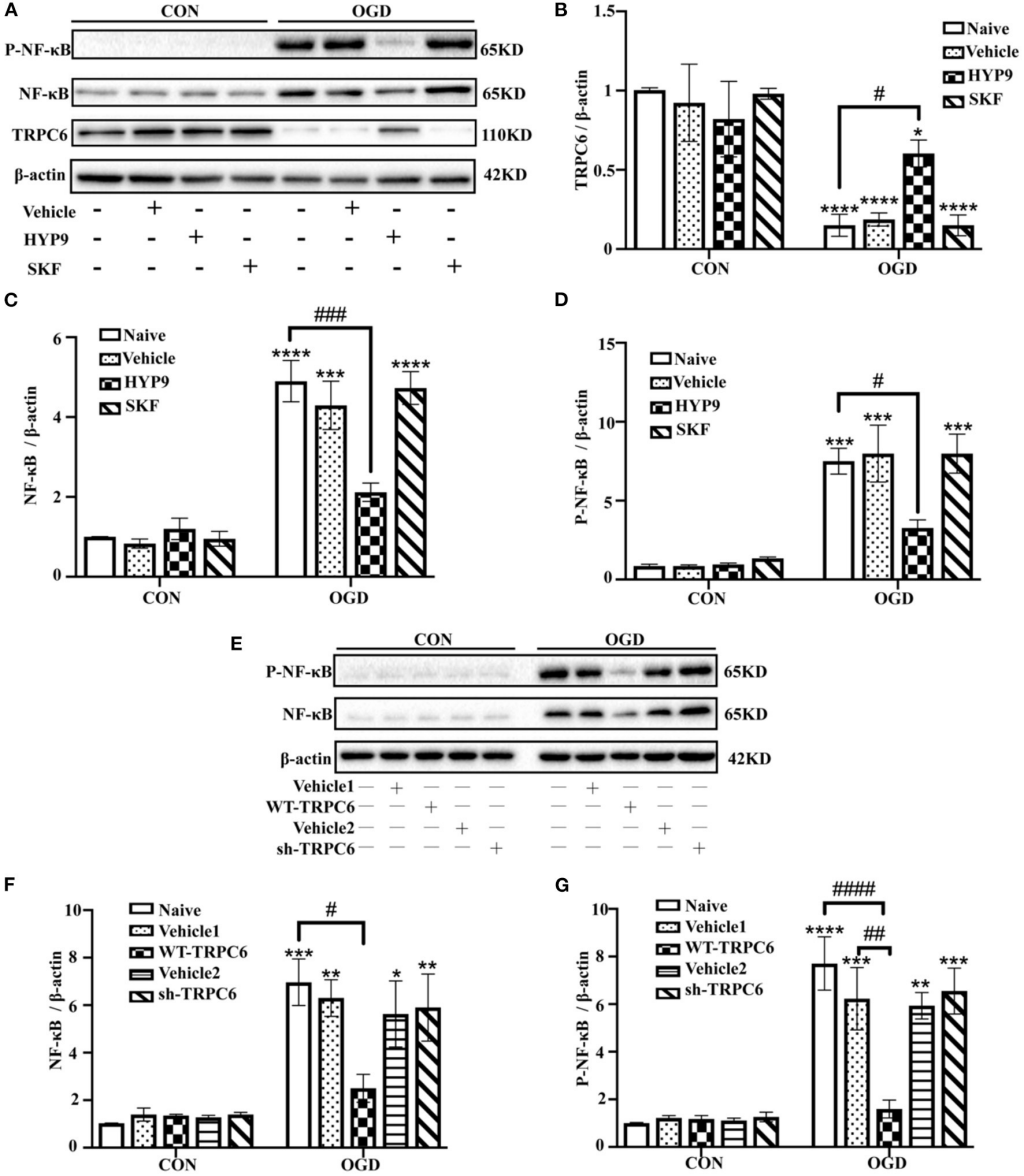
TRPC6 diminishes OGD/R-induced NF-κB phosphorylation in astrocytes. Astrocytes were pre-incubated with Vehicle (DMSO, the final concentration is 0.025%), 15 μM HYP9 or 30 μM SKF for 12 h, and then expose to control condition (CON) or 3 h OGD and 24 h reperfusion (OGD). **(A)** Immunoblots for TRPC6, NF-κB, and phosphorylated-NF-κB (P-NF-κB) of the extracts from cortical astrocytes. β-actin was used as a loading control. **(B–D)** Quantification of TRPC6, NF-κB, and P-NF-κB protein levels shown in **(A)**. Data were shown as mean ± SEM (*n* = 3). Astrocytes were infected with lentiviruses carrying Vehicle1, WT-TRPC6, Vehicle2, or sh-TRPC6, and then exposed to control condition (CON) or 3 h OGD and 24 h reperfusion (OGD). **(E)** Immunoblots for NF-κB and P-NF-κB of the extracts from cortical astrocytes. β-actin was used as a loading control. **(F,G)** Quantification of NF-κB, and P-NF-κB protein levels shown in **(E)**. Data were shown as mean ± SEM (*n* = 3). *p < 0.05, **p < 0.01, ***p < 0.001, ****p < 0.0001 vs. CON + Naive group; ^#^*p* < 0.05, ^##^*p* < 0.01, ^###^*p* < 0.001, ^####^*p* < 0.0001 vs. OGD + Naive or OGD + Vehicle1 group.

NF-κB phosphorylation and nuclear translocation are considered vital pathways that mediate the inflammatory cascade, including cytokine generation (Kopitar-Jerala, 2015; Shih et al., 2015). Therefore, the influence of HYP9 and SKF on NF-κB nuclear translocation and phosphorylation in primary mouse astrocytes exposed to ischemic damage was investigated. Immunofluorescent signals showed the nuclear distribution of NF-κB was increased after OGD/R (Figure 9). Notably, the TRPC6-specific activator HYP9 attenuated IR injury-induced NF-κB nuclear translocation (Figure 9). Furthermore, HYP9 and WT-TRPC6 inhibited the phosphorylation of NF-κB in astrocytes subjected to OGD/R (Figures 10A–G). Collectively, the findings indicate that NF-κB phosphorylation and nuclear translocation may be pivotal downstream pathways that contribute to the protective effects of TRPC6 in astrocytes after stroke.

## Discussion

In cerebral ischemia, several clinical trials have failed due to narrow therapeutic window, adverse effects, and individual differences (Petrovic-Djergovic et al., 2016). A breakthrough in stroke therapeutic strategies is considered urgent. In the present study, significant downregulation of TRPC6 protein was observed in both cultured astrocytes and cerebral cortices after IR injury. *In vivo* results confirmed that the TRPC6 channel-specific activator HYP9 attenuated the infarct volume, astrocytes population, apoptosis, and the generation of pro-inflammatory cytokines (IL-6 and IL-1β) caused by MCAO. However, significant effects of SKF on infarct lesion or cortical IL-6 and IL-1β release in MCAO mice were not observed. In addition, HYP9 was shown to attenuate apoptosis rate, improve cell vitality, and reduce the generation of IL-6 and IL-1β in cultured astrocytes subjected to OGD/R. Furthermore, the TRPC channel antagonist SKF aggravated astrocytic apoptosis and cytotoxicity in OGD groups. In addition, inhibition of the TRPC6 channel downregulation with HYP9 suppressed the increase in [Ca^2+^]_i_ in ischemic astrocytes. The IR-mediated increased NF-κB phosphorylation and nuclear translocation were inhibited by HYP9 pretreatment in primary mouse astrocytes. In parallel, overexpression of TRPC6 also decreased IR injury-induced astrocytic apoptosis, cytotoxicity, inflammatory responses and NF-κB phosphorylation in modeled ischemia *in vitro*.

Previously, neuron-specific-related mechanisms are primarily considered in the pathogenesis of brain ischemia. As knowledge increased, astrocytes and other CNS cells are shown to be closely involved in ischemic stroke (Pekny et al., 2016; Werner et al., 2020). Astrocytes interact with neurons and participate in structural support, neuronal metabolism, synaptogenesis, synaptic transmission, axonal remodeling, and neurogenesis (Halassa and Haydon, 2010; Liu and Chopp, 2016). IR injury-activated astrocytes initiate the CNS inflammatory response, which in turn exacerbates brain insults during ischemic stroke (Cekanaviciute and Buckwalter, 2016). Thus, maintaining the normal function of astrocytes likely promotes post-ischemic neurological recovery. Therapeutic targeting of astrocytes is expected to be a future area of research regarding treatment and prognosis of brain ischemia (Neuhaus et al., 2017).

Evidence shows that TRPC6 is a crucial regulator of inflammatory cascades, especially in pulmonary inflammation (Chen et al., 2020; Ortiz-Muñoz et al., 2020). TRPC6 contributes to platelet activation, leukocyte transendothelial migration, lung vascular barrier disruption, and airway inflammation (Tauseef et al., 2012; Weber et al., 2015; Chen et al., 2020; Ortiz-Muñoz et al., 2020). In addition, activation of the TRPC6 channel promotes apoptosis in neonatal glomerular mesangial cells and renal tubular epithelial cells under different injury conditions (Soni and Adebiyi, 2016; Hou et al., 2018). TRPC6 inhibits N-methyl-D-aspartate (NMDA)-mediated Ca^2+^ elevation and reduces neuronal ischemic excitotoxity (Li et al., 2012). Furthermore, inhibition of TRPC6 degradation in neurons through cAMP-response element binding protein (CREB) pathway alleviates ischemic cerebral insults (Du et al., 2010). (-)-Epigallocatechin-3-gallate, resveratrol, and neuroprotectin D1 contribute to neuroprotective effect on brain IR damage through TRPC6/CREB pathways (Lin et al., 2013b; Yao et al., 2013, 2014). In particular, Hyperforin, a selective TRPC6 agonist, alleviates cerebral IR injury by suppressing the degradation of TRPC6 and increasing phosphorylated CREB in Ca^2+^/calmodulin-dependent kinase IV (CaMKIV) signaling pathway (Lin et al., 2013a). Collectively, these evidences suggest the vital role of TRPC6/CREB pathway in ischemic stroke. Studies have shown that TRPC6 channel is expressed in microglia and involved in microglial activation and neuroinflammatory response in different diseases including hypertension, epilepsy, Alzheimer's disease, infectious diseases and neuropathic pain (Toth et al., 2013; Lee et al., 2014; Liu et al., 2017; Shinjyo et al., 2020; Wang et al., 2020b). However, the relevance of the TRPC6 channel in IR injury-induced astrocytic apoptosis and inflammation remains unclear. In addition, other downstream mechanisms beyond TRCP6/CREB pathways are worth exploring.

Hyperforin, a main compound of St. John's wort, can activate the TRPC6 channel and induce changes in [Ca^2+^]_i_ currents (Leuner et al., 2007); however, hyperforin is chemically unstable when subjected to oxygen and light. HYP9 is a stable synthetic phloroglucinol derivative of hyperforin that maintains the biological function of hyperforin in activating the TRPC6 channel (Leuner et al., 2010). Consequently, HYP9 was used in the present study to explore its function in the TRPC6 channel and the associated downstream cellular mechanisms in ischemic stroke. Inflammatory responses and apoptosis in ischemic astrocytes were inhibited by maintaining the protein level of the TRPC6 channel *via* HYP9. Overexpression of TRPC6 also reduced IR injury-induced astrocytic apoptosis, cytotoxicity, and inflammatory responses in experimental stroke *in vitro*. Meanwhile, SKF is a TRPC channel blocker (Song et al., 2014; Jing et al., 2016). SKF also affects other channels belonging to the TRPC channel subfamily. Evidence shows that TRPC1, TRPC3, and TRPC7 are involved in cerebral ischemic stroke (Chen et al., 2017; Xu et al., 2018). In the present study, SKF aggravated astrocyte injury after ischemia *in vitro*. However, significant changes in infarct volume, cleaved caspase-3 expression, and cytokine release were not observed in the MCAO model after SKF application. The inconsistent results between cell and animal models of stroke may be due to the complex effects of SKF on cortical non-astrocytic cells. Noticeably, knocking down TRPC6 via sh-TRPC6 shown no significant effects on apoptosis, cytotoxicity, and inflammatory responses in primary astrocytes after OGD/R. Since TRPC6 is strikingly downregulated in astrocytes exposed to IR insults, knocking down of TRPC6 did not significantly aggravate astrocytic damage in OGD group. The dissimilar effects of SKF and sh-TRPC6 on ischemic astrocytes might be related to the side-effect of SKF on other TRPCs (TRPC3/7). Overall, further *in vivo* studies using astrocytic TRPC6-, siRNA-, or shRNA-targeted transgenic mice are needed to fully clarify the role of the astrocytic TRPC6 channel in stroke.

Activation of the cerebral inflammatory cascade has important roles in acute brain IR insults. The inflammatory response induced by IR injury directly affect the progress of cerebral ischemia (Petrovic-Djergovic et al., 2016; Ramiro et al., 2018). Notably, astrocytes regulate the neuroinflammatory response by affecting the generation and release of diverse cytokines in ischemic stroke (Sofroniew, 2014). Astrocytes generate several pro-inflammatory cytokines and chemokines, including IL-6, IL-1β, tumor necrosis factor-α (TNF-α), C-C motif chemokine ligand (CCL)-2, CCL-5, CCL-7, C-X-C motif chemokine ligand (CXCL)-10, and vasoactive endothelial growth factor (John et al., 2005; Hamby et al., 2012; Sofroniew, 2015). Astrocyte-derived IL-6 and IL-1β are essential for secondary inflammatory damage in brain ischemia (John et al., 2005; Diaz-Cañestro et al., 2019). The pro-inflammatory nuclear transcription factor NF-κB belongs to the detrimental astrocytic signaling pathways (Colombo and Farina, 2016). NF-κB is involved in astrocyte inflammation and apoptosis (Su et al., 2019; Bai et al., 2020). Compelling evidence has shown that inhibition of NF-κB nuclear translocation or phosphorylation alleviates IR-mediated cytokine production and brain inflammation (Colombo and Farina, 2016; Liu et al., 2019; Xie et al., 2019; Bai et al., 2020). NF-κB activation is tightly modulated by various typical upstream signaling molecules, which include toll-like receptors, TNF receptors, IL-1 receptors, CAMKII, and stromal interaction molecule or Orai-mediated Ca^2+^ signals (Verstrepen et al., 2008; Berry et al., 2018; Pires et al., 2018; Ye et al., 2019).

A crucial relationship exists between Ca^2+^ and NF-κB signaling pathways (Wei et al., 2019). As a vital second messenger, Ca^2+^ governs a variety of functions causing cellular pathological and physiological changes. Modulation of the Ca^2+^ current has attracted interest as a promising medical strategy for ischemic stroke (Kalogeris et al., 2016; Secondo et al., 2018). The TRPC6 channel, a Ca^2+^ permeable cation channel, has been associated with Ca^2+^ entry and brain ischemic pathogenesis (Liu et al., 2020). In the present study, TRPC6 was dramatically downregulated during IR injury. The TRPC6-specific agonist HYP9 rescued TRPC6 degradation in primary cultured astrocytes and reduced [Ca^2+^]_i_. Consistently, the TRPC channel antagonist SKF contributed to increased [Ca^2+^]_i_. How a Ca^2+^ entry activator reduces and a Ca^2+^ channel blocker increases the Ca^2+^ influx in primary astrocytes is notable. TRPC6 has been shown to block the NMDA receptor (NMDAR)-triggered [Ca^2+^]_i_ increase and neurotoxicity in neurons after brain IR damage (Li et al., 2012). Overexpression of TRPC6 inhibits [Ca^2+^]_i_ overload, prevents neuronal death, lessen infarct size, and improves behavior performance after ischemia. Similarly, another research team suggests that the TRPC6 channel selectively suppresses the NMDAR-mediated current in hippocampal neurons, which is increased *via* TRPC6 knockdown or interference with SKF (Shen et al., 2013). In another study, SKF increases intracellular Ca^2+^ by promoting the reverse mode of the Na^+^/Ca^2+^ exchanger (Song et al., 2014). Thus, we hypothesized that the underlying mechanism of TRPC6-induced [Ca^2+^]_i_ decrease in astrocytes after ischemic insults may be associated with the complex interaction between the TRPC6 channel and other ion exchangers.

Although the present study showed a protective role of the TRPC6 channel in astrocytes after ischemia, various questions remain unanswered. The effects of the astrocytic TRPC6 channel on ischemic neurons will be addressed in our future work. Furthermore, the underlying mechanisms of TRPC6-triggered astrocytic Ca^2+^ changes in ischemic stroke warrant further investigations.

## Conclusion

The results of the present study indicate that HYP9 reduces the infarct size, apoptosis, and pro-inflammatory cytokine release in a mouse model of ischemic stroke. Inhibition of TRPC6 channel downregulation *via* HYP9 or TRPC6 overexpression alleviates apoptosis and inflammatory response in astrocytes exposed to ischemic damage. Furthermore, the protective role of TRPC6 in stroke is associated with Ca^2+^/NF-κB-dependent pathways. The results of this study indicate that the astrocytic TRPC6 channel and TRPC6 agonist HYP9 might be a novel therapeutic approach to prevent ischemic stroke induced by brain injury.

## Data Availability Statement

The original contributions presented in the study are included in the article/supplementary materials, further inquiries can be directed to the corresponding author/s.

## Ethics Statement

The animal study was reviewed and approved by Animal Care Committee of the First Affiliated Hospital at Zhejiang University.

## Author Contributions

LL wrote the manuscript. LL, MC, and KL finished the experiment. SZ and XXio designed the general idea. All authors edited the drafts of the manuscript.

## Conflict of Interest

The authors declare that the research was conducted in the absence of any commercial or financial relationships that could be construed as a potential conflict of interest.

## References

[B1] Bai X. Zhang Y. L. Liu L. N. (2020). Inhibition of TRIM8 restrains ischaemia-reperfusion-mediated cerebral injury by regulation of NF-κB activation associated inflammation and apoptosis. Exp. Cell Res. 388:111818. 10.1016/j.yexcr.2020.111818 31917201

[B2] Berry C. T. May M. J. Freedman B. D. (2018). STIM- and Orai-mediated calcium entry controls NF-κB activity and function in lymphocytes. Cell Calcium 74, 131–143. 10.1016/j.ceca.2018.07.003 30048879 PMC6415950

[B3] Cekanaviciute E. Buckwalter M. S. (2016). Astrocytes: integrative regulators of neuroinflammation in stroke and other neurological diseases. Neurotherapeutics 13, 685–701. 10.1007/s13311-016-0477-8 27677607 PMC5081110

[B4] Chen Q. Zhou Y. Zhou L. Fu Z. Yang C. Zhao L. (2020). TRPC6-dependent Ca(2+) signaling mediates airway inflammation in response to oxidative stress via ERK pathway. Cell Death Dis. 11:170 10.1038/s41419-020-2360-0 32139669 PMC7058000

[B5] Chen X. Lu M. He X. Ma L. Birnbaumer L. Liao Y. (2017). TRPC3/6/7 knockdown protects the brain from cerebral ischemia injury via astrocyte apoptosis inhibition and effects on NF-κB translocation. Mol. Neurobiol. 54, 7555–7566. 10.1007/s12035-016-0227-2 27826749

[B6] Chen X. Taylor-Nguyen N. N. Riley A. M. Herring B. P. White F. A. Obukhov A. G. (2019). The TRPC6 inhibitor, larixyl acetate, is effective in protecting against traumatic brain injury-induced systemic endothelial dysfunction. J. Neuroinflamm. 16:21. 10.1186/s12974-019-1407-6 30704505 PMC6354413

[B7] Colombo E. Farina C. (2016). Astrocytes: key regulators of neuroinflammation. Trends Immunol. 37, 608–620. 10.1016/j.it.2016.06.006 27443914

[B8] Curcic S. Tiapko O. Groschner K. (2019). Photopharmacology and opto-chemogenetics of TRPC channels-some therapeutic visions. Pharmacol. Ther. 200, 13–26. 10.1016/j.pharmthera.2019.04.003 30974125

[B9] Deng Y. L. Ma Y. L. Zhang Z. L. Zhang L. X. Guo H. Qin P.. (2018). Astrocytic N-Myc downstream-regulated gene-2 is involved in nuclear transcription factor κB-mediated inflammation induced by global cerebral ischemia. Anesthesiology 128, 574–586. 10.1097/aln.0000000000002044 29252510

[B10] Diaz-Cañestro C. Reiner M. F. Bonetti N. R. Liberale L. Merlini M. Wüst P.. (2019). AP-1 (activated protein-1) transcription factor JunD regulates ischemia/reperfusion brain damage via IL-1β (interleukin-1β). Stroke 50, 469–477. 10.1161/strokeaha.118.023739 30626291

[B11] Ding X. He Z. Zhou K. Cheng J. Yao H. Lu D.. (2010). Essential role of TRPC6 channels in G2/M phase transition and development of human glioma. J. Natl. Cancer Inst. 102, 1052–1068. 10.1093/jnci/djq217 20554944

[B12] Du W. Huang J. Yao H. Zhou K. Duan B. Wang Y. (2010). Inhibition of TRPC6 degradation suppresses ischemic brain damage in rats. J. Clin. Invest. 120, 3480–3492. 10.1172/jci43165 20811149 PMC2947234

[B13] Griesi-Oliveira K. Acab A. Gupta A. R. Sunaga D. Y. Chailangkarn T. Nicol X.. (2015). Modeling non-syndromic autism and the impact of TRPC6 disruption in human neurons. Mol. Psychiatry 20, 1350–1365. 10.1038/mp.2014.141 25385366 PMC4427554

[B14] Guo C. Ma Y. Ma S. Mu F. Deng J. Duan J.. (2017). The role of TRPC6 in the neuroprotection of calycosin against cerebral ischemic injury. Sci. Rep. 7:3039. 10.1038/s41598-017-03404-6 28596571 PMC5465205

[B15] Halassa M. M. Haydon P. G. (2010). Integrated brain circuits: astrocytic networks modulate neuronal activity and behavior. Annu. Rev. Physiol. 72, 335–355. 10.1146/annurev-physiol-021909-135843 20148679 PMC3117429

[B16] Hamby M. E. Coppola G. Ao Y. Geschwind D. H. Khakh B. S. Sofroniew M. V. (2012). Inflammatory mediators alter the astrocyte transcriptome and calcium signaling elicited by multiple G-protein-coupled receptors. J. Neurosci. 32, 14489–14510. 10.1523/jneurosci.1256-12.2012 23077035 PMC3518872

[B17] Hamid R. Newman J. H. (2009). Evidence for inflammatory signaling in idiopathic pulmonary artery hypertension: TRPC6 and nuclear factor-kappaB. Circulation 119, 2297–2298. 10.1161/circulationaha.109.855197 19414653

[B18] Hankey G. J. (2017). Stroke. Lancet 389, 641–654. 10.1016/s0140-6736(16)30962-x 27637676

[B19] Hou X. Xiao H. Zhang Y. Zeng X. Huang M. Chen X.. (2018). Transient receptor potential channel 6 knockdown prevents apoptosis of renal tubular epithelial cells upon oxidative stress via autophagy activation. Cell Death Dis. 9:1015. 10.1038/s41419-018-1052-5 30282964 PMC6170481

[B20] Jia Y. Zhou J. Tai Y. Wang Y. (2007). TRPC channels promote cerebellar granule neuron survival. Nat. Neurosci. 10, 559–567. 10.1038/nn1870 17396124

[B21] Jing Z. Sui X. Yao J. Xie J. Jiang L. Zhou Y.. (2016). SKF-96365 activates cytoprotective autophagy to delay apoptosis in colorectal cancer cells through inhibition of the calcium/CaMKIIγ/AKT-mediated pathway. Cancer Lett. 372, 226–238. 10.1016/j.canlet.2016.01.006 26803057 PMC5240807

[B22] John G. R. Lee S. C. Song X. Rivieccio M. Brosnan C. F. (2005). IL-1-regulated responses in astrocytes: relevance to injury and recovery. Glia 49, 161–176. 10.1002/glia.20109 15472994

[B23] Kalogeris T. Baines C. P. Krenz M. Korthuis R. J. (2016). Ischemia/reperfusion. Compr. Physiol. 7, 113–170. 10.1002/cphy.c160006 28135002 PMC5648017

[B24] Khoshnam S. E. Winlow W. Farzaneh M. Farbood Y. Moghaddam H. F. (2017). Pathogenic mechanisms following ischemic stroke. Neurol. Sci. 38, 1167–1186. 10.1007/s10072-017-2938-1 28417216

[B25] Kim Y. J. Kang T. C. (2015). The role of TRPC6 in seizure susceptibility and seizure-related neuronal damage in the rat dentate gyrus. Neuroscience 307, 215–230. 10.1016/j.neuroscience.2015.08.054 26327362

[B26] Kopitar-Jerala N. (2015). Innate immune response in brain, NF-kappaB signaling and cystatins. Front. Mol. Neurosci. 8:73. 10.3389/fnmol.2015.00073 26696821 PMC4673337

[B27] Lee S. K. Kim J. E. Kim Y. J. Kim M. J. Kang T. C. (2014). Hyperforin attenuates microglia activation and inhibits p65-Ser276 NFκB phosphorylation in the rat piriform cortex following status epilepticus. Neurosci. Res. 85, 39–50. 10.1016/j.neures.2014.05.006 24881563

[B28] Leuner K. Heiser J. H. Derksen S. Mladenov M. I. Fehske C. J. Schubert R.. (2010). Simple 2,4-diacylphloroglucinols as classic transient receptor potential-6 activators–identification of a novel pharmacophore. Mol. Pharmacol. 77, 368–377. 10.1124/mol.109.057513 20008516

[B29] Leuner K. Kazanski V. Müller M. Essin K. Henke B. Gollasch M.. (2007). Hyperforin–a key constituent of St. John's wort specifically activates TRPC6 channels. FASEB J. 21, 4101–4111. 10.1096/fj.07-8110com 17666455

[B30] Li H. Huang J. Du W. Jia C. Yao H. Wang Y. (2012). TRPC6 inhibited NMDA receptor activities and protected neurons from ischemic excitotoxicity. J. Neurochem. 123, 1010–1018. 10.1111/jnc.12045 23043486

[B31] Li H. Xie Y. Zhang N. Yu Y. Zhang Q. Ding S. (2015). Disruption of IP3R2-mediated Ca^2+^ signaling pathway in astrocytes ameliorates neuronal death and brain damage while reducing behavioral deficits after focal ischemic stroke. Cell Calcium 58, 565–576. 10.1016/j.ceca.2015.09.004 26433454 PMC4658295

[B32] Li Q. Cao Y. Dang C. Han B. Han R. Ma H.. (2020). Inhibition of double-strand DNA-sensing cGAS ameliorates brain injury after ischemic stroke. EMBO Mol. Med. 12:e11002. 10.15252/emmm.201911002 32239625 PMC7136961

[B33] Li Y. Jia Y. C. Cui K. Li N. Zheng Z. Y. Wang Y. Z.. (2005). Essential role of TRPC channels in the guidance of nerve growth cones by brain-derived neurotrophic factor. Nature 434, 894–898. 10.1038/nature03477 15758952

[B34] Lin Y. Chen F. Zhang J. Wang T. Wei X. Wu J.. (2013b). Neuroprotective effect of resveratrol on ischemia/reperfusion injury in rats through TRPC6/CREB pathways. J. Mol. Neurosci. 50, 504–513. 10.1007/s12031-013-9977-8 23435869

[B35] Lin Y. Zhang J. C. Fu J. Chen F. Wang J. Wu Z. L.. (2013a). Hyperforin attenuates brain damage induced by transient middle cerebral artery occlusion (MCAO) in rats via inhibition of TRPC6 channels degradation. J. Cereb. Blood Flow Metab. 33, 253–262. 10.1038/jcbfm.2012.164 23149561 PMC3564196

[B36] Liu H. Wu X. Luo J. Wang X. Guo H. Feng D.. (2019). Pterostilbene attenuates astrocytic inflammation and neuronal oxidative injury after ischemia-reperfusion by inhibiting NF-κB phosphorylation. Front. Immunol. 10:2408. 10.3389/fimmu.2019.02408 31681297 PMC6811521

[B37] Liu L. Gu L. Chen M. Zheng Y. Xiong X. Zhu S. (2020). Novel targets for stroke therapy: special focus on TRPC channels and TRPC6. Front. Aging Neurosci. 12:70. 10.3389/fnagi.2020.00070 32256338 PMC7093711

[B38] Liu N. Zhuang Y. Zhou Z. Zhao J. Chen Q. Zheng J. (2017). NF-κB dependent up-regulation of TRPC6 by Aβ in BV-2 microglia cells increases COX-2 expression and contributes to hippocampus neuron damage. Neurosci. Lett. 651, 1–8. 10.1016/j.neulet.2017.04.056 28458019

[B39] Liu Z. Chopp M. (2016). Astrocytes, therapeutic targets for neuroprotection and neurorestoration in ischemic stroke. Prog. Neurobiol. 144, 103–120. 10.1016/j.pneurobio.2015.09.008 26455456 PMC4826643

[B40] Montell C. (2001). Physiology, phylogeny, and functions of the TRP superfamily of cation channels. Sci. STKE 2001:re1. 10.1126/stke.2001.90.re1 11752662

[B41] Neuhaus A. A. Couch Y. Hadley G. Buchan A. M. (2017). Neuroprotection in stroke: the importance of collaboration and reproducibility. Brain 140, 2079–2092. 10.1093/brain/awx126 28641383

[B42] Ortiz-Muñoz G. Yu M. A. Lefrançais E. Mallavia B. Valet C. Tian J. J.. (2020). Cystic fibrosis transmembrane conductance regulator dysfunction in platelets drives lung hyperinflammation. J. Clin. Invest. 130, 2041–2053. 10.1172/jci129635 31961827 PMC7108932

[B43] Pekny M. Pekna M. Messing A. Steinhäuser C. Lee J. M. Parpura V.. (2016). Astrocytes: a central element in neurological diseases. Acta Neuropathol. 131, 323–345. 10.1007/s00401-015-1513-1 26671410

[B44] Petrovic-Djergovic D. Goonewardena S. N. Pinsky D. J. (2016). Inflammatory disequilibrium in stroke. Circ. Res. 119, 142–158. 10.1161/circresaha.116.308022 27340273 PMC5138050

[B45] Pires B. R. B. Silva R. Ferreira G. M. Abdelhay E. (2018). NF-kappaB: two sides of the same coin. Genes (Basel) 9:24. 10.3390/genes9010024 29315242 PMC5793177

[B46] Pochwat B. Szewczyk B. Kotarska K. Rafało-Ulińska A. Siwiec M. Sowa J. E.. (2018). Hyperforin potentiates antidepressant-like activity of lanicemine in mice. Front. Mol. Neurosci. 11:456. 10.3389/fnmol.2018.00456 30618608 PMC6299069

[B47] Qu Z. Wang Y. Li X. Wu L. Wang Y. (2017). TRPC6 expression in neurons is differentially regulated by NR2A- and NR2B-containing NMDA receptors. J. Neurochem. 143, 282–293. 10.1111/jnc.14215 28902407

[B48] Quick K. Zhao J. Eijkelkamp N. Linley J. E. Rugiero F. Cox J. J.. (2012). TRPC3 and TRPC6 are essential for normal mechanotransduction in subsets of sensory neurons and cochlear hair cells. Open Biol. 2:120068. 10.1098/rsob.120068 22724068 PMC3376737

[B49] Rakers C. Petzold G. C. (2017). Astrocytic calcium release mediates peri-infarct depolarizations in a rodent stroke model. J. Clin. Invest. 127, 511–516. 10.1172/jci89354 27991861 PMC5272189

[B50] Ramirez G. A. Coletto L. A. Sciorati C. Bozzolo E. P. Manunta P. Rovere-Querini P.. (2018). Ion channels and transporters in inflammation: special focus on TRP channels and TRPC6. Cells 7:70. 10.3390/cells7070070 29973568 PMC6070975

[B51] Ramiro L. Simats A. García-Berrocoso T. Montaner J. (2018). Inflammatory molecules might become both biomarkers and therapeutic targets for stroke management. Ther. Adv. Neurol. Disord. 11:1756286418789340. 10.1177/1756286418789340 30093920 PMC6080077

[B52] Riccio A. Medhurst A. D. Mattei C. Kelsell R. E. Calver A. R. Randall A. D.. (2002). mRNA distribution analysis of human TRPC family in CNS and peripheral tissues. Brain Res. Mol. Brain Res. 109, 95–104. 10.1016/s0169-328x(02)00527-2 12531519

[B53] Secondo A. Bagetta G. Amantea D. (2018). On the role of store-operated calcium entry in acute and chronic neurodegenerative diseases. Front. Mol. Neurosci. 11:87. 10.3389/fnmol.2018.00087 29623030 PMC5874322

[B54] Shen H. Pan J. Pan L. Zhang N. (2013). TRPC6 inhibited NMDA current in cultured hippocampal neurons. Neuromol. Med. 15, 389–395. 10.1007/s12017-013-8226-1 23494294

[B55] Shen Y. Qin H. Chen J. Mou L. He Y. Yan Y.. (2016). Postnatal activation of TLR4 in astrocytes promotes excitatory synaptogenesis in hippocampal neurons. J. Cell Biol. 215, 719–734. 10.1083/jcb.201605046 27920126 PMC5147000

[B56] Shih R. H. Wang C. Y. Yang C. M. (2015). NF-kappaB signaling pathways in neurological inflammation: a mini review. Front. Mol. Neurosci. 8:77. 10.3389/fnmol.2015.00077 26733801 PMC4683208

[B57] Shinjyo N. Nakayama H. Li L. Ishimaru K. Hikosaka K. Suzuki N.. (2020). Hypericum perforatum extract and hyperforin inhibit the growth of neurotropic parasite *Toxoplasma gondii* and infection-induced inflammatory responses of glial cells *in vitro*. J. Ethnopharmacol. 113525. 10.1016/j.jep.2020.113525. [Epub ahead of print]. 33129946

[B58] Shirakawa H. Katsumoto R. Iida S. Miyake T. Higuchi T. Nagashima T.. (2017). Sphingosine-1-phosphate induces Ca(2+) signaling and CXCL1 release via TRPC6 channel in astrocytes. Glia 65, 1005–1016. 10.1002/glia.23141 28300348

[B59] Singh A. Hildebrand M. E. Garcia E. Snutch T. P. (2010). The transient receptor potential channel antagonist SKF96365 is a potent blocker of low-voltage-activated T-type calcium channels. Br. J. Pharmacol. 160, 1464–1475. 10.1111/j.1476-5381.2010.00786.x 20590636 PMC2938817

[B60] Sofroniew M. V. (2014). Multiple roles for astrocytes as effectors of cytokines and inflammatory mediators. Neuroscientist. 20, 160–172. 10.1177/1073858413504466 24106265

[B61] Sofroniew M. V. (2015). Astrocyte barriers to neurotoxic inflammation. Nat. Rev. Neurosci. 16, 249–263. 10.1038/nrn3898 25891508 PMC5253239

[B62] Song M. Chen D. Yu S. P. (2014). The TRPC channel blocker SKF 96365 inhibits glioblastoma cell growth by enhancing reverse mode of the Na(+)/Ca(2+) exchanger and increasing intracellular Ca(2+). Br. J. Pharmacol. 171, 3432–3447. 10.1111/bph.12691 24641279 PMC4105931

[B63] Soni H. Adebiyi A. (2016). TRPC6 channel activation promotes neonatal glomerular mesangial cell apoptosis via calcineurin/NFAT and FasL/Fas signaling pathways. Sci Rep. 6:29041. 10.1038/srep29041 27383564 PMC4935859

[B64] Su Y. Zong S. Wei C. Song F. Feng H. Qin A.. (2019). Salidroside promotes rat spinal cord injury recovery by inhibiting inflammatory cytokine expression and NF-κB and MAPK signaling pathways. J. Cell Physiol. 234, 14259–14269. 10.1002/jcp.28124 30656690 PMC13483101

[B65] Tauseef M. Knezevic N. Chava K. R. Smith M. Sukriti S. Gianaris N. (2012). TLR4 activation of TRPC6-dependent calcium signaling mediates endotoxin-induced lung vascular permeability and inflammation. J. Exp. Med. 209, 1953–1968. 10.1084/jem.20111355 23045603 PMC3478927

[B66] Toth P. Tucsek Z. Sosnowska D. Gautam T. Mitschelen M. Tarantini S.. (2013). Age-related autoregulatory dysfunction and cerebromicrovascular injury in mice with angiotensin II-induced hypertension. J. Cereb. Blood Flow Metab. 33, 1732–1742. 10.1038/jcbfm.2013.143 23942363 PMC3824186

[B67] Venkatachalam K. Montell C. (2007). TRP channels. Annu. Rev. Biochem. 76, 387–417. 10.1146/annurev.biochem.75.103004.142819 17579562 PMC4196875

[B68] Verstrepen L. Bekaert T. Chau T. L. Tavernier J. Chariot A. Beyaert R. (2008). TLR-4, IL-1R and TNF-R signaling to NF-kappaB: variations on a common theme. Cell Mol. Life Sci. 65, 2964–2978. 10.1007/s00018-008-8064-8 18535784 PMC11131687

[B69] Wang H. Cheng X. Tian J. Xiao Y. Tian T. Xu F.. (2020a). TRPC channels: structure, function, regulation and recent advances in small molecular probes. Pharmacol. Ther. 209:107497. 10.1016/j.pharmthera.2020.107497 32004513 PMC7183440

[B70] Wang J. Zhao M. Jia P. Liu F. F. Chen K. Meng F. Y.. (2020b). The analgesic action of larixyl acetate, a potent TRPC6 inhibitor, in rat neuropathic pain model induced by spared nerve injury. J. Neuroinflamm. 17:118. 10.1186/s12974-020-01767-8 32299452 PMC7164269

[B71] Weber E. W. Han F. Tauseef M. Birnbaumer L. Mehta D. Muller W. A. (2015). TRPC6 is the endothelial calcium channel that regulates leukocyte transendothelial migration during the inflammatory response. J. Exp. Med. 212, 1883–1899. 10.1084/jem.20150353 26392222 PMC4612081

[B72] Wei T. Wang Y. Xu W. Liu Y. Chen H. Yu Z. (2019). KCa3.1 deficiency attenuates neuroinflammation by regulating an astrocyte phenotype switch involving the PI3K/AKT/GSK3β pathway. Neurobiol. Dis. 132:104588. 10.1016/j.nbd.2019.104588 31470105

[B73] Werner Y. Mass E. Ashok Kumar P. Ulas T. Händler K. Horne A.. (2020). Cxcr4 distinguishes HSC-derived monocytes from microglia and reveals monocyte immune responses to experimental stroke. Nat. Neurosci. 23, 351–362. 10.1038/s41593-020-0585-y 32042176 PMC7523735

[B74] Xie W. Zhu T. Dong X. Nan F. Meng X. Zhou P.. (2019). HMGB1-triggered inflammation inhibition of notoginseng leaf triterpenes against cerebral ischemia and reperfusion injury via MAPK and NF-κB signaling pathways. Biomolecules 9:512. 10.3390/biom9100512 31547018 PMC6843331

[B75] Xu N. Meng H. Liu T. Feng Y. Qi Y. Wang H. (2018). TRPC1 deficiency exacerbates cerebral ischemia/reperfusion-induced neurological injury by potentiating Nox4-derived reactive oxygen species generation. Cell Physiol. Biochem. 51, 1723–1738. 10.1159/000495676 30504729

[B76] Yao C. Zhang J. Chen F. Lin Y. (2013). Neuroprotectin D1 attenuates brain damage induced by transient middle cerebral artery occlusion in rats through TRPC6/CREB pathways. Mol. Med. Rep. 8, 543–550. 10.3892/mmr.2013.1543 23799606

[B77] Yao C. Zhang J. Liu G. Chen F. Lin Y. (2014). Neuroprotection by (-)-epigallocatechin-3-gallate in a rat model of stroke is mediated through inhibition of endoplasmic reticulum stress. Mol. Med. Rep. 9, 69–76. 10.3892/mmr.2013.1778 24193141

[B78] Ye J. Das S. Roy A. Wei W. Huang H. Lorenz-Guertin J. M.. (2019). Ischemic injury-induced CaMKIIδ and CaMKIIγ confer neuroprotection through the NF-κB signaling pathway. Mol. Neurobiol. 56, 2123–2136. 10.1007/s12035-018-1198-2 29992531 PMC6394630

[B79] Zhang H. Sun S. Wu L. Pchitskaya E. Zakharova O. Fon Tacer K.. (2016). Store-operated calcium channel complex in postsynaptic spines: a new therapeutic target for Alzheimer's disease treatment. J. Neurosci. 36, 11837–11850. 10.1523/jneurosci.1188-16.2016 27881772 PMC5125243

[B80] Zhang Y. Qin W. Zhang L. Wu X. Du N. Hu Y.. (2015). MicroRNA-26a prevents endothelial cell apoptosis by directly targeting TRPC6 in the setting of atherosclerosis. Sci. Rep. 5:9401. 10.1038/srep09401 25801675 PMC4371083

[B81] Zhou J. Du W. Zhou K. Tai Y. Yao H. Jia Y.. (2008). Critical role of TRPC6 channels in the formation of excitatory synapses. Nat. Neurosci. 11, 741–743. 10.1038/nn.2127 18516035

